# [M^II^(H_2_dapsc)]-[Cr(CN)_6_] (M = Mn, Co) Chain and Trimer Complexes: Synthesis, Crystal Structure, Non-Covalent Interactions and Magnetic Properties

**DOI:** 10.3390/molecules27238518

**Published:** 2022-12-03

**Authors:** Valentina D. Sasnovskaya, Leokadiya V. Zorina, Sergey V. Simonov, Artem D. Talantsev, Eduard B. Yagubskii

**Affiliations:** 1 Institute of Problems of Chemical Physics RAS, Federal Research Center of Problems of Chemical Physics and Medical Chemistry RAS, Chernogolovka 142432, Russia; 2 Institute of Solid State Physics RAS, Chernogolovka 142432, Russia

**Keywords:** crystal structure, non-covalent interactions, seven-coordinate PBP complexes, dc and ac magnetic properties, spin-glass, metamagnetic transition, field-induced single-molecule magnetism

## Abstract

Four new heterometallic complexes combining [M^II^(H_2_dapsc)]^2+^ cations with the chelating H_2_dapsc {2,6-diacetylpyridine-bis(semicarbazone)} Schiff base ligand and [Cr(CN)_6_]^3−^ anion were synthesized: {[M^II^(H_2_dapsc)]Cr^III^(CN)_6_K(H_2_O)_2.5_(EtOH)_0.5_}*_n_*·1.2*n*(H_2_O), M = Mn (**1**) and Co (**2**), {[Mn(H_2_dapsc)]_2_Cr(CN)_6_(H_2_O)_2_}Cl·H_2_O (**3**) and {[Co(H_2_dapsc)]_2_Cr(CN)_6_(H_2_O)_2_}Cl·2EtOH·3H_2_O (**4**). In all the compounds, M(II) centers are seven-coordinated by N_3_O_2_ atoms of H_2_dapsc in the equatorial plane and N or O atoms of two apical –CN/water ligands. Crystals **1** and **2** are isostructural and contain infinite negatively charged chains of alternating [M^II^(H_2_dapsc)]^2+^ and [Cr^III^(CN)_6_]^3−^ units linked by CN-bridges. Compounds **3** and **4** consist of centrosymmetric positively charged trimers in which two [M^II^(H_2_dapsc)]^2+^ cations are bound through one [Cr^III^(CN)_6_]^3−^ anion. All structures are regulated by π-stacking of coplanar H_2_dapsc moieties as well as by an extensive net of hydrogen bonding. Adjacent chains in **1** and **2** interact also by coordination bonds via a pair of K^+^ ions. The compounds containing Mn^II^ (**1**, **3**) and Co^II^ (**2**, **4**) show a significant difference in magnetic properties. The ac magnetic measurements revealed that complexes **1** and **3** behave as a spin glass and a field-induced single-molecule magnet, respectively, while **2** and **4** do not exhibit slow magnetic relaxation in zero and non-zero dc fields. The relationship between magnetic properties and non-covalent interactions in the structures **1**–**4** was traced.

## 1. Introduction

Molecular nanomagnets, the so-called single-molecular magnets (SMMs), single-chain magnets (SCMs) and single-ion magnets (SIMs) are attracting much attention due to their unique magnetic properties, such as superparamagnetism, relaxation, blocking and quantum tunneling magnetization [1,2,3], as well as the prospects for their application in information technology, spintronics and quantum calculations [4,5,6]. However, at present, the practical use of molecular nanomagnets is difficult, since they have low critical magnetization blocking temperatures (*T_B_*), below which their unique magnetic properties appear. Although, in recent times, SIMs, mononuclear metallocene complexes of Dy, with blocking temperatures close to liquid nitrogen temperature have been synthesized [7,8], their application is unlikely since these compounds are extremely air-sensitive and have short magnetization relaxation times. For instance, for SIM ([(Cp^iPr5^)Dy(Cp*)]^+^ with *T_B_* = 80 K, long relaxation times (τ > 100 s) occur only below ~30 K [8]. In 2021, Tang et al. reported an air-stable hexagonal bipyramidal Dy(III) SMM, [Dy(L^N6^)(Ph_3_SiO)_2_][PF_6_], which displayed a record anisotropy barrier (*U*_eff_) exceeding 1800 K (*T_B_* = 20 K) and the longest relaxation time approaching 2500 s at 2.0 K for all known air-stable SIMs [9]. However, it is difficult to expect that sufficiently long magnetization relaxation times can be achieved with mononuclear complexes. Recently, a new concept for the design of advanced molecular nanomagnets has been proposed based on the use of low-spin (S = 1/2) pentagonal-bipyramidal complexes of 4d^3^ and 5d^3^ metals as building blocks for the synthesis of M(4d/5d)–M(3d) exchange-coupled pairs with Ising-type exchange interactions [10].

**Scheme 1:**
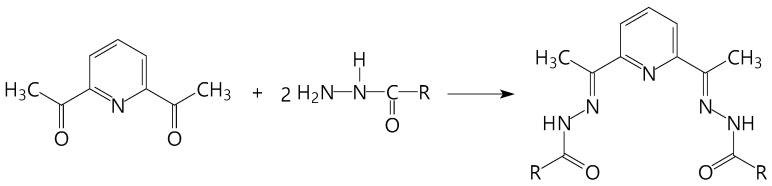
Some diacetylpyridine-based acyclic pentadentate (N_3_O_2_) ligands: R = NH_2_ (H_2_dapsc), 2-OHC_6_H_4_ (H_4_daps); 4-OCH_3_C_6_H_4_ (H_2_dapmbh), C_6_H_5_ (H_2_dapbh), C_6_H_5_-C_6_H_4_ (Biph), CH_3_CHOH (*l*-daplh).

Increasing the magnetization reversal barriers (*U*_eff_) and blocking temperatures (*T_B_*∞*U*_eff_) for molecular nanomagnets are associated, on the one hand, with using the metal ions that have large uniaxial magnetic anisotropy (4d, 5d, 4f and some 3d elements) [3,11,12,13] and, on the other hand, with coordination environment of the metal centers [11,12,13,14,15,16,17,18,19,20,21]. The coordination geometry around the metal essentially affects its local magnetic anisotropy [14,17,18,19,20,21]. Experimental and theoretical studies of seven-coordinated pentagonal-bipyramidal (PBP) complexes show that PBP geometry of a coordination polyhedron contributes to an increase in magnetic anisotropy of the metal center [14,22,23,24,25,26,27,28]. In this regard, of considerable interest is the family of acyclic pentadentate (N_3_O_2_) ligands (Scheme 1), which are widely used for the targeted synthesis of PBP complexes of 3d, and more recently, 4d, 5d and 4f metals [10,29,30,31,32,33,34,35,36,37,38,39,40,41,42,43,44,45,46,47,48]. Their PBP geometry results from the pentagonal coordination around the metal ion of nearly planar N_3_O_2_ ligand in the equatorial plane and two apical labile ligands (usually solvent molecules and/or various simple anions). Moreover, depending on the synthesis conditions, these ligands can be in neutral, monoanionic or dianionic forms, which leads to a wide variety of these complexes. The study of the magnetic properties of mononuclear PBP complexes of this family showed that some of them (Fe^II^, Co^II^, Dy and Er) are field-induced SIMs [22,23,40,43,45,46,47,48]. It should be noted that the Co PBP complexes have a planar magnetic anisotropy (+D), in contrast to similar complexes of Fe(II) and Ni(II), which have an axial Ising-type anisotropy (–D) [23,24,26,46,47,49].

The presence of labile ligands in the axial positions of the PBP complexes makes them attractive building blocks for the construction of low-dimensional heteronuclear compounds by replacing these ligands with bridging cyanometallates or the PBP complexes with cyanide apical groups [17,23,24,37,49,50,51,52,53,54,55,56]. Up to date, tri-, penta-, and decanuclear SMMs and coordination polymeric SCMs have been obtained along the way.

Earlier, we studied the reactions of [Mn(H_2_dapsc)Cl_2_]·H_2_O (dapsc=2,6-diacetylpyridine bis(semicarbazone), Scheme 1) with K_3_[Mn(CN)_6_] and K_3_[Fe(CN)_6_] and obtained the chain polymeric complexes {[Mn(H_2_dapsc)][Mn(CN)_6_][K(H_2_O)_2.75_(MeOH)_0.5_]}*_n_*·0.5*n*(H_2_O) and {[Mn(H_2_dapsc)][Fe(CN)_6_][K(H_2_O)_3.5_]}*_n_*·1.5*n*H_2_O, which revealed single-chain magnet (SCM) behavior [50,52].

In this work, we investigated the reactions of complexes [Mn(H_2_dapsc)Cl_2_]·H_2_O and [Co(H_2_dapsc)Cl(H_2_O)]Cl·H_2_O with K_3_[Cr(CN)_6_] and (Ph_4_P)_3_[Cr(CN)_6_]. As a result, four cyano-bridged heterometallic complexes were synthesized: {[M(H_2_dapsc)]Cr(CN)_6_K(H_2_O)_2.5_(EtOH)_0.5_}*_n_*·1.2*n*(H_2_O), M = Mn (**1**) and Co (**2**); and {[Mn(H_2_dapsc)]_2_Cr(CN)_6_(H_2_O)_2_}Cl·H_2_O (**3**) and {[Co(H_2_dapsc)]_2_Cr(CN)_6_(H_2_O)_2_}Cl·2EtOH·3H_2_O (**4**). Their crystal structures and magnetic properties have been studied. Herein, we present these results.

## 2. Results and Discussion

### 2.1. Synthesis and Crystal Structure

The reactions of [Mn(H_2_dapsc)Cl_2_]·H_2_O (**5**) and [Co(H_2_dapsc)ClH_2_O]Cl·2H_2_O (**6**) with K_3_[Cr(CN)_6_] and (Ph_4_P)_3_Cr(CN)_6_ were studied. The interaction of **5** and **6** with K_3_[Cr(CN)_6_] in ethanol/water led to the formation of the chain polymeric complexes **1** and **2**, respectively. The use of (Ph_4_P)_3_Cr(CN)_3_ instead of K_3_[Cr(CN)_6_] gave linear trinuclear complexes **3** and **4**, see Materials and Methods.

**Figure 1:**
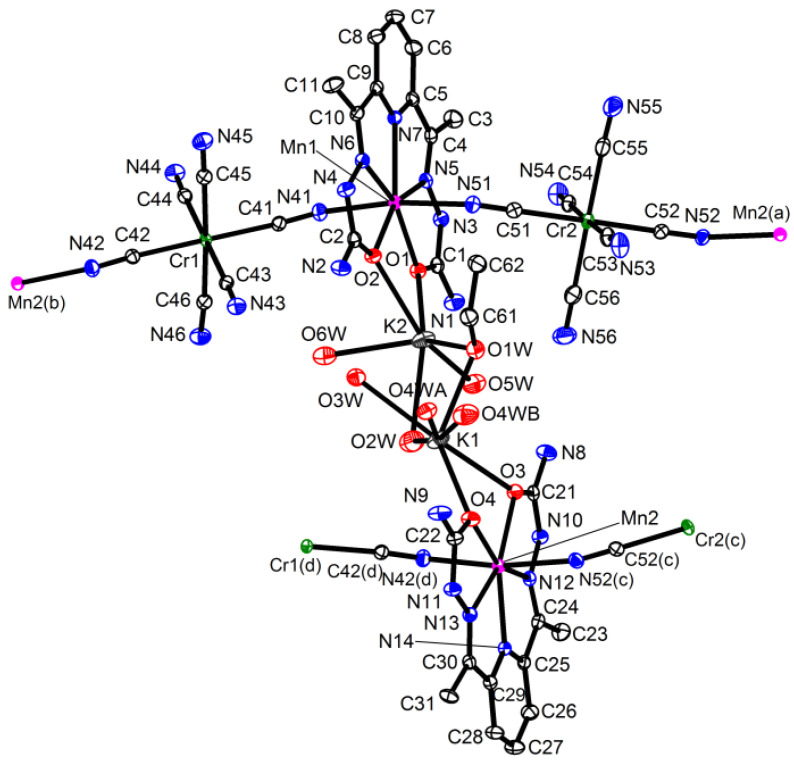
Asymmetric unit in **1** with atom numbering scheme (ORTEP drawing with 50% probability ellipsoids, solvate water molecules are omitted for clarity). Colored atoms: Cr (green), K (grey), Mn (magenta), N (blue), O (red). Symmetry codes: (a) (−*x*, *y* − 0.5, −*z*), (b) (1 − *x*, *y* − 0.5, 1 − *z*), (c) (−*x*, *y* + 0.5, −*z*) and (d) (1 − *x*, *y* + 0.5, 1 − *z*).

{[M(H_2_dapsc)]Cr(CN)_6_K(H_2_O)_2.5_(EtOH)_0.5_}*_n_*·1.2*n*(H_2_O), M = Mn (**1**) or Co (**2**). The complexes **1** and **2** are isostructural and crystallize in the monoclinic *P*2_1_ space group. The asymmetric unit includes two [M^II^(H_2_dapsc)], two [Cr(CN)_6_] moieties, two K^+^ ions, one EtOH solvent and eight water molecules, all in general positions. Two water molecules (O8w, O9w) have 0.7 occupancy; one water (O4w) is disordered between two sites with an occupancy ratio of 0.8/0.2 in **1** and 0.6/0.4 in **2**. An ORTEP drawing of **1** is shown in Figure 1, and the key bond distances and angles in **1** and **2** are listed in Table A1 of the Appendix A section.

**Figure 2:**
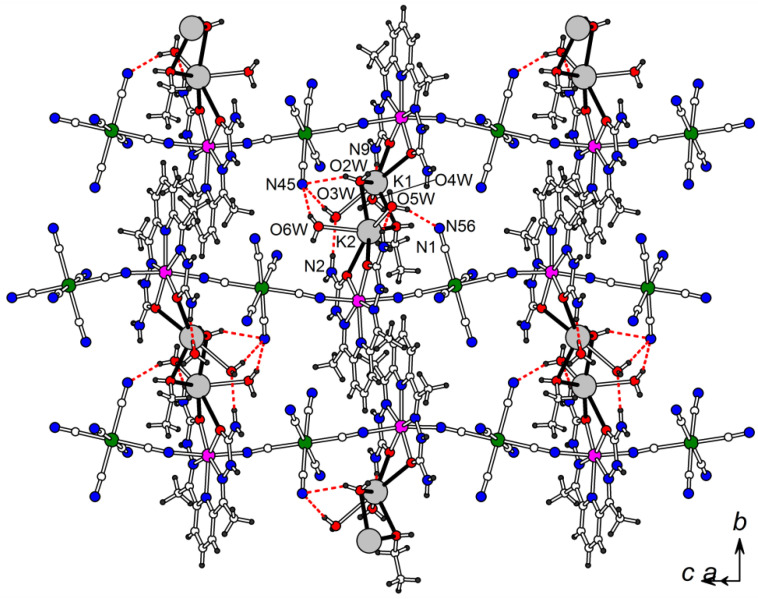
Infinite CN-bridged {[M^II^(H_2_dapsc)]^2+^[Cr^III^(CN)_6_]^3−^}*_n_* chains in **1** interacting by π-π stacking of H_2_dapsc ligands and coordination bonds via K^+^/O in the (1 0 –1) plane. Interchain coordination bonds via pairs of K^+^ cations are shown by black bonds. Hydrogen bonds are shown by red dashed lines (see Tables S1 and S2 for hydrogen bond geometry in **1** and **2**, respectively).

The crystal structure contains infinite negatively charged chains of alternating cationic [M^II^(H_2_dapsc)]^2+^ and anionic [Cr^III^(CN)_6_]^3−^ units running along [1 0 1] (Figure 2). The metal centers along the chain are connected through the CN-linkage. The chain is bent due to non-linear M(II)-N-C angles (150–152° in **1** and 155–161° in **2**), whereas the Cr-C-N angles are closer to 180° (173–176° in **1** and 170–173° in **2**, Table A1). The M(II) ions have a pentagonal bipyramidal coordination geometry and are surrounded by two O and three N atoms from H_2_dapsc in the equatorial plane and two axial N atoms from CN-bridges. The M-O,N bond lengths in the [M^II^(H_2_dapsc)] moieties are shorter in the Co(II) compound **2** compared with the Mn(II) compound **1**, the maximal difference ~0.1 Å being observed for M-N bonds (Table A1). Both independent [M^II^(H_2_dapsc)] fragments in **1** and **2** are almost flat: the dihedral angle between the two semicarbazone planes of the H_2_dapsc ligand defined by seven non-metallic atoms of two pentagonal cycles [for example, O1, C1, N3, N5, C4, C5, N7 and O2, C2, N4, N6, C10, C9, N7] is 1.48(4)° for Mn(1), 1.22(4)° for Mn(2), 3.05(5)° for Co(1) and 2.58(4)° for Co(2). The Cr-C_CN_ distances in the anionic units are in the range of 2.06-2.08 Å.

The compounds **1** and **2** include K^+^ ions compensating for the negative charge of the {[M^II^(H_2_dapsc)]^2+^[Cr^III^(CN)_6_]^3−^}*_n_* chain. K^+^ is coordinated with oxygen atoms of M^II^(H_2_dapsc) at the K-O distances of 2.713(2)–2.783(2) Å in **1** and 2.721(3)–2.752(3) Å in **2**. The ethanol molecule and five of the eight independent water molecules O(2w)-O(6w) also belong to the coordination sphere of K^+^ ions; the corresponding K-O distances are 2.73–2.84 Å. Two K^+^ cations linked to [M(H_2_dapsc)] from adjacent chains are at the K-K distance of 4.154(1) and 4.165(1) Å in **1** and **2**, respectively, and bridged together through two oxygen atoms from one water and one ethanol molecule. The coordination bonds via the pairs of K^+^ ions join adjacent infinite chains in the (1 0 –1) plane (Figure 2). Additionally, the chains are fixed together by non-covalent interactions: by π-π stacking of the nearest H_2_dapsc ligands as well as by hydrogen bonds N−H_H2dapsc_···O_water_, O−H_water_···N_anion_ in the (1 0 –1) plane and N−H_H2dapsc_...N_anion_ in the (0 1 0) plane (Figure 2 and Figure S1 in the Supplementary Materials, hydrogen bond geometry is given in Tables S1 and S2).

**Figure 3:**
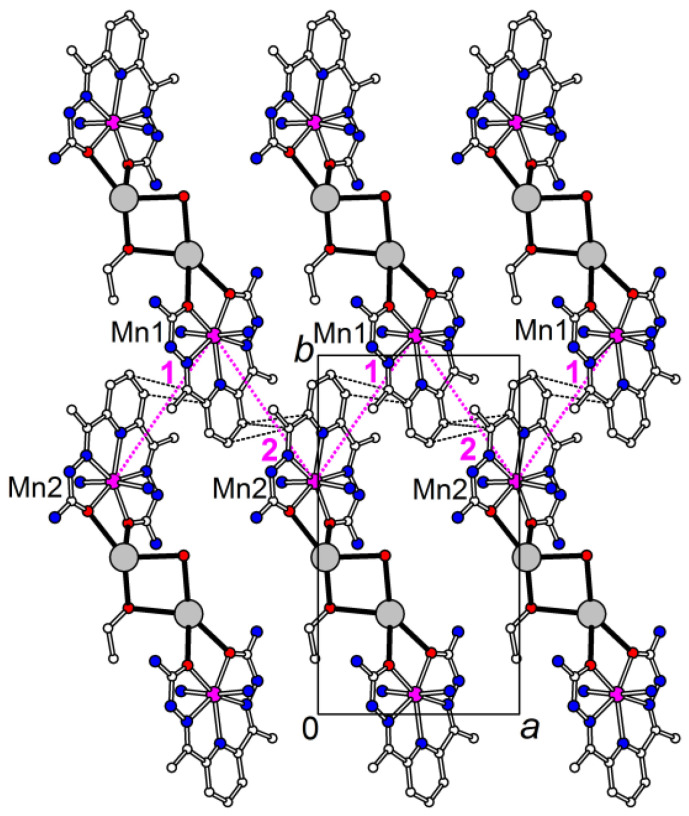
Part of the structure **1** displaying zigzag infinite chain of π-stacked coplanar [Mn(H_2_dapsc)] units; the [Cr(CN)_6_] anions, H atoms and most of water molecules are omitted for clarity. The Mn-Mn distances are 8.2341(6) and 8.2357(6) Å for 1 and 2 interactions, respectively (magenta dotted lines). Shortened C…C contacts (<3.6 Å) between H_2_dapsc fragments are shown by black dashed lines.

The Mn^II^-Cr^III^ distances along the chain are 5.2292(5), 5.2404(5) for Mn(1) and 5.2190(5), 5.2340(6) Å for Mn(2). The Co^II^-Cr^III^ chain is more compact, the corresponding Co^II^-Cr^III^ distances are 5.1579(7), 5.1703(7) for Co(1) and 5.1570(7), 5.1903(7) Å for Co(2). The shortest interchain intermetallic distances are found between the M^II^(1) and M^II^(2) centers connected via π-π stacking of dapsc ligands: 8.2341(6) and 8.2357(6) Å in the Mn^II^ structure and 8.0793(7) and 8.1262(7) Å in the Co^II^ structure. The π-stacked coplanar [M(II)(H_2_dapsc)] units in both the **1** and **2** structures form infinite zigzag chains along the *a*-direction with almost uniform M(II)-M(II) separations (Figure 3).

The complexes **1** and **2** are isostructural to the other chain complex {[Mn(H_2_dapsc)][Mn(CN)_6_][K(H_2_O)_2.75_(MeOH)_0.5_]}*_n_*·0.5*n*(H_2_O) (**7**) [50]. The main difference between them is another solvent composition and the presence of disorder in the K^+^ site in **7**. The chain complex {[Mn(H_2_dapsc)][Fe(CN)_6_][K(H_2_O)_3.5_]}*_n_*·1.5*n*H_2_O (**8**) [52] also has similar unit cell parameters but higher symmetry (*P*2_1_/*n*) because the pair of K^+^ ions in **8** are bridged by two water molecules related by inversion symmetry instead of water/ethanol in **1** and **2** or water/methanol in **7**.

**Figure 4:**
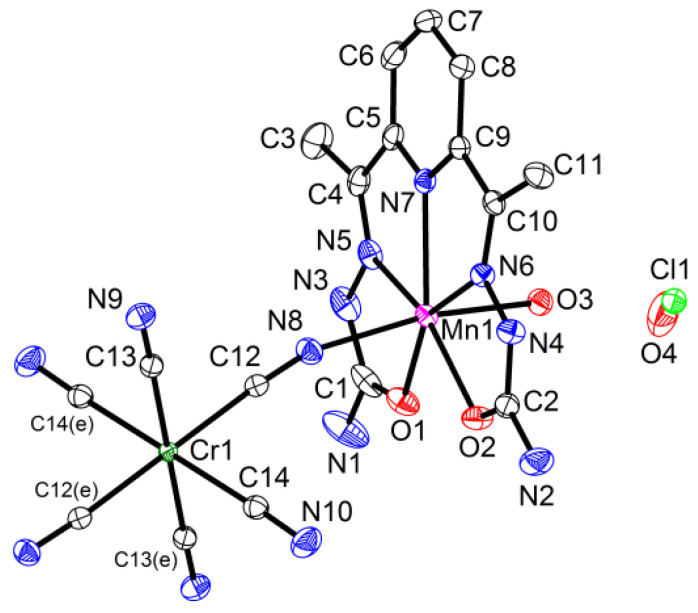
Asymmetric unit in **3** with atom numbering scheme (ORTEP drawing with 50% probability ellipsoids). Colored atoms: Cl (light green), Cr (green), Mn (magenta), N (blue), O (red). Symmetry code: (e) (1 − *x*, −*y*, −*z*).

{[Mn(H_2_dapsc)]_2_Cr(CN)_6_(H_2_O)_2_}Cl·H_2_O (**3**). Complex **3** crystallizes in the monoclinic *P*2_1_/*c* space group. The asymmetric unit includes half of the formula unit with Cr(1) atom in the inversion center; a half-occupied Cl^−^ anion and solvate water are mixed in the close sites. An ORTEP drawing of **3** is shown in Figure 4, and the key bond distances and angles are listed in Table A1.

**Figure 5:**
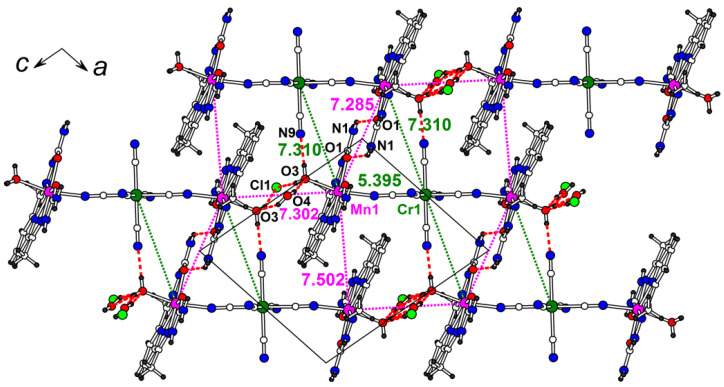
The ac layer in the structure **3**. Intermetallic distances (in Å) are shown (magenta color for Mn…Mn, green color for Mn…Cr). Hydrogen bonds are shown by red dashed lines (see Table S3 for hydrogen bond geometry).

Structure **3** is built from centrosymmetric positively charged trimers {H_2_O-[Mn(H_2_dapsc)]^2+^-[Cr(CN)_6_]^3−^-[Mn(H_2_dapsc)]^2+^-H_2_O}^+^ elongated along [1 0 –1] (Figure 5). Mn(II) and Cr(III) ions are linked through the *trans*-CN ligands of [Cr(CN)_6_]; the Mn-N-C angle is 164.7(1)°, the Mn-Cr distance is 5.3952(3) Å. The Mn(II) center has a distorted pentagonal bipyramidal environment of two oxygen and three nitrogen atoms of the equatorial H_2_dapsc ligand and axial N, O atoms from CN-bridge and terminal H_2_O ligand. The H_2_dapsc ligand is flattened: the dihedral angle between two semicarbazone planes defined by seven non-metallic atoms of two pentagonal cycles of H_2_dapsc (as for the structure **1**) is 4.55(4)°. 

The non-covalent intermolecular interactions are well represented in the structure **3**. The trimers are connected with each other and with the anion/water site by hydrogen bonding (red dashed lines in Figure 5 and Table S3 for details). The shortest intermetallic distances are found in the ac plane. The adjacent [Mn(H_2_dapsc)] units with the smallest Mn-Mn distance, 7.2853(5) Å, interact through a pair of strong N-H…O bonds formed between -NH_2_ function and oxygen atoms of H_2_dapsc ligands. The nearby Mn-Cr distance is comparable (7.3100(4) Å) and the additional O-H_H2O_…N_anion_ hydrogen bond is formed. The terminal water molecules linked to Mn are hydrogen bonded to the similar water terminal of another trimer via the Cl^−^/H_2_O pair, the Mn…Mn distance in this interaction is 7.3022(5) Å. The π-π stacking in pairs of Mn(H_2_dapsc) moieties from adjacent trimers is also observed (Mn-Mn is 7.5018(5) Å, Figure 5).

**Figure 6:**
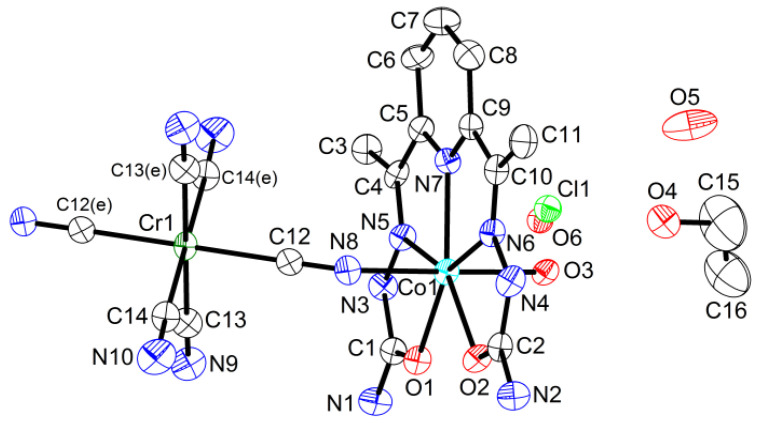
Asymmetric unit in **4** with atom numbering scheme (ORTEP drawing with 50% probability ellipsoids). Colored atoms: Cl (light green), Co (cyan), Cr (green), N (blue), O (red). Symmetry code: (e) (1 − *x*, −*y*, −*z*).

{[Co(H_2_dapsc)]_2_Cr(CN)_6_(H_2_O)_2_}Cl·2EtOH·3H_2_O (**4**). Complex **4** crystallizes in the triclinic *P*$\bar{1}$ space group. The asymmetric unit includes half of the formula unit with the Cr(1) atom in the inversion center; the half-occupied Cl^−^ and water are mixed in the close sites. An ORTEP drawing of **4** is shown in Figure 6, and the key bond distances and angles are listed in Table A1.

Structure **4** contains centrosymmetric positively charged trimers like structure **3**, although these compounds are not isostructural. The Co-Cr-Co trimer in **4** is more compact than the Mn-Cr-Mn trimer in **3** due to the smaller ionic radius of Co(II) (0.72 Å) in comparison with Mn(II) (0.82 Å) and the stronger bending of the Co chain (the Co-N-C angle is 151.3(4)°). The Co-Cr distance in the trimer is 5.1281(9) Å. The dihedral angle between the two semicarbazone planes in the H_2_dapsc ligand is 1.2(1)°.

**Figure 7:**
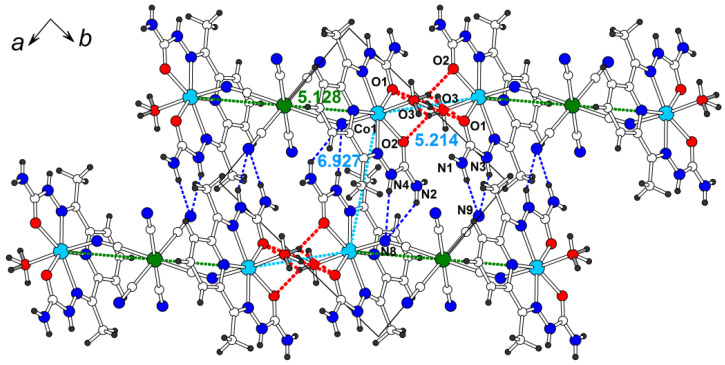
The *ab* layer in the structure **4** (solvate water and ethanol are omitted for clarity). Intermetallic distances (in Å) are shown (cyan color for Co…Co, green color for Co…Cr). Hydrogen O-H…O/N-H…N bonds are shown by red/blue dashed lines (see Table S4 for hydrogen bond geometry).

The shortest intermetallic distances are observed for the non-covalently interacting trimers in the *ab* plane (Figure 7). A pair of strong O-H_H2O_…O_H2dapsc_ hydrogen bonds (red dashed lines in Figure 7 and Table S4 for details) are observed between adjacent trimers along the [1 −1 0] direction whose terminal water molecules are close to each other. The Co-Co distance in this contact, 5.214(1) Å, is very short and only slightly larger than the Co-Cr distance inside the trimer. For this reason, it is possible to consider the trimers interacting in this way as an infinite chain of the [-Co(H_2_dapsc)-(2H_2_O)-Co(H_2_dapsc)-Cr(CN)_6_-]_∞_ composition. The Co-Co distance between two [Co(H_2_dapsc)] moieties interacting by π-stacking is 6.927(1) Å (Figure 7). The N−H_H2dapsc_...N_anion_ hydrogen bonds are also formed in the *ab* plane (blue dashed lines in Figure 7). The presence of mixed H_2_O/Cl^−^ position causes a disorder in the hydrogen atoms attached to the water and ethanol molecules.

### 2.2. Magnetic Properties

#### 2.2.1. Static (dc) Magnetic Properties

**Figure 8:**
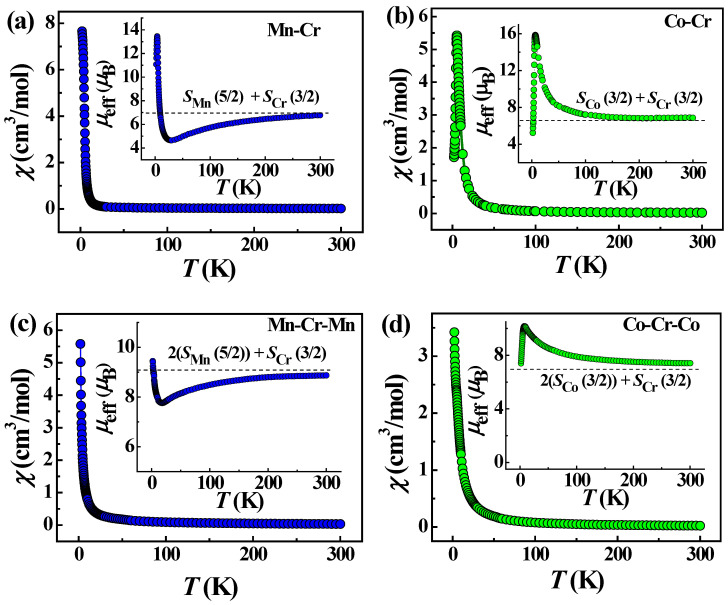
Temperature dependencies of magnetic susceptibility χ_mol_ and effective magnetic moment μ_eff_ (insets) for **1** (**a**), **2** (**b**), **3** (**c**) and **4** (**d**). The blue and green colors of the symbols distinguish the data for Mn- and Co-based complexes, respectively. The dashed lines on the insets show the estimations of effective magnetic moment for a unit cell with the Co, Cr and Mn spins, indicated on the legends.

Variable-temperature magnetic measurements were performed on polycrystalline samples of complexes **1**–**4** in the range of 1.8–300 K under a dc field of 1000 Oe. The plots of temperature dependencies of the magnetic susceptibility (χ_M_) and effective magnetic moment (μ_eff_) for the isostructural chain complexes **1** and **2** are depicted in Figure 8a,b.

The μ_eff_ value at 300 K for **1** is close to the expected value (7.06 μ_B_) for non-interacting Mn(II) and Cr(III) ions with S = 5/2 (g = 2.0) and 3/2 (g = 2.0), respectively. For **1**, at cooling, the μ_eff_ value gradually decreases down to 50 K, and then, it decreases rapidly to the minimum value at ∼20 K. Upon further lowering the temperature, the μ_eff_ increases abruptly to reach a maximum at 3 K, followed by a slight drop to 1.8 K. The latter feature is probably attributed to the presence of antiferromagnetic (AF) coupling between the chains through the hydrogen bonding network, π−π stacking and the interchain coordination bonds via solvent molecules and K^+^ ions. The general behavior of the μ_eff_ with temperature suggests AF exchange interactions between Mn(II) and Cr(III) spin carriers within the chains, resulting in a ferrimagnetic spin arrangement along the chains. The Curie–Weiss fit of the magnetic susceptibility data above 75 K gave a Weiss constant of −75.0 K (Figure S2, Table S5). The large negative Weiss constant confirms the dominance of AF interactions in the chains of complex **1**. The rapid growth of μ_eff_ below 20 K indicates a ferrimagnetic ordering in the chains of **1**.

**Figure 9:**
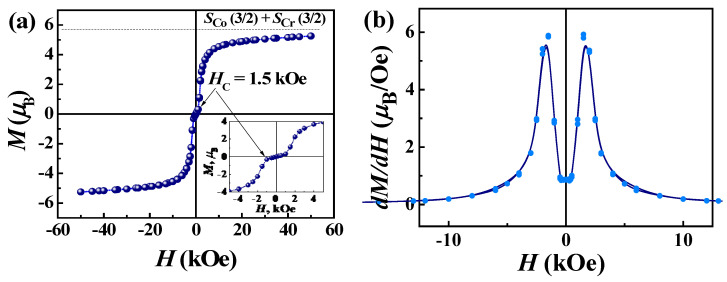
M(H) loop (**a**) and its first derivative (**b**) at 2 K for complex **2**.

The magnetic properties of complex **2** differ significantly from those of **1**. The μ_eff_ value at room temperature is higher than the spin-only value (5.47 μ_B_) for one Co(II) of S = 3/2 (g = 2.0) and one Cr(III) of S = 3/2 (g = 2.0) due to the orbital contributions of Co(II) ions, Figure 8b. As the temperature decreases, the effective magnetic moment does not decrease, as in the case of **1**, but, on the contrary, increases to a sharp maximum around 6.0 K, indicating an intrachain ferromagnetic coupling between the Co(II) and Cr(III) ions, which is expected for pseudolinear Co(II)-NC-Cr(III) bridges [49,51,57,58]. The Weiss constant is positive and amounts to 7.0 K (Figure S2, Table S5), which confirms the presence of ferromagnetic interactions in the chains of complex **2**. X-ray diffraction analysis of **1** and **2** showed that the Co-Cr chains are more compact; the Co-Cr distances are ~0.07 Å shorter than the Mn-Cr distances. Below 6 K, the value of the effective magnetic moment for **2** shows a sharp and deep drop to 1.8 K, which is probably due to the presence of intrinsic magnetic anisotropy of the Co(II) centers [59] and/or antiferromagnetic ordering associated with coupling between chains through non-covalent interactions. The χ_M_ vs. T behavior also exhibits a maximum (Figure 8b), suggesting antiferromagnetic ordering. The interchain AF interactions in structure **2** are much stronger than in **1**. The shortest interchain distance between Co(1) and Co(2) centers linked through π-π-stacking of H_2_dapsc ligands is 0.15 Å shorter than the Mn(1)–Mn(2) distance in **1**. The study of the field-dependent magnetization at 2 K indicates a metamagnetic-like transition from an AF state to a state of spontaneous magnetization under the field H_c_ = 1.5 kOe, Figure 9a. The dM/dH(H) dependence exhibits distinct peaks in the fields 1.5 kOe and −1/5 kOe, Figure 9b. The metamagnetic nature of the transition is also evidenced by the character of the magnetization curves M(T) and the curves χ_mol(T)_ recorded at different magnetic fields (Figure S3) as well as the data of ac magnetic susceptibility. The field dependence of the real part (χ’) of ac susceptibility shows a maximum of χ’ at a field of 1.5 kOe (Figure S4). The application of an external constant magnetic field leads to the suppression of interchain AF coupling and causes a metamagnetic transition. Such transitions have been observed in other heterospin as well as homospin compounds with interchain AF ordering [52,60,61,62,63,64,65,66,67].

Unlike **1** and **2**, the trinuclear complexes **3** and **4** are not isostructural. Their magnetic properties differ markedly and, in some respects, are close to those of the corresponding chain complexes. The temperature dependencies of the magnetic susceptibility (χ_M_) and effective magnetic moment (μ_eff_) for the Mn_2_Cr trimer **3** are depicted in Figure 8c. The μ_eff_ value at 300 K is close to the expected value (9.2 μ_B_) for two non-interacting Mn(II) and one Cr(III) ions. Upon cooling from room temperature, the μ_eff_ value decreases to approximately 16 K, as in the case of complex **1**, indicating antiferromagnetic interaction between the metallic centers, as was observed in the similar trimer [68]. On further cooling, the μ_eff_ increases to 1.8 K. Unlike **1**, this increase is not accompanied by a subsequent decrease in μ_eff_ associated with AF interactions between the chains in structure **1**. Theoretical analysis of the antiferromagnetic interactions between the Mn^II^(H_2_dapsc) units belonging to the neighboring chains, which contact through π-π stacking of planar H_2_dapsc ligands and a system of hydrogen bonds in the complex {[Mn(H_2_dapsc)][Fe(CN)_6_][K(H_2_O)_3.5_]}*_n_*·1.5*n*H_2_O, showed that contacts through π-π stacking play a decisive role in these superexchange AF interactions [52]. In the structure **1**, along the *a*-axis, there are infinite chains of ...-Mn1-Mn2-Mn1-Mn2-... through π-π stacking of the H_2_dapsc ligands with distances between metals of 8.2341(6) and 8.2357(6) Å (Figure 3), whereas in **3,** such contacts are present only in pairs (Figure 5). Probably for this reason, interchain AF interactions in **3** are very weak compared to those in **1**.

Temperature behavior of μ_eff_ for the Co_2_Cr trimer (**4**) (Figure 8d) is close to that for the chain complex (-Cr-CN-Co-NC-)*_n_* (**2**), Figure 8b. Upon cooling, the μ_eff_ value increases, reaching a maximum near 7.8 K, which indicates a ferromagnetic exchange between the metal centers in the trimer, and then decreases. The Weiss constant is positive and equal to 27.0 K (Figure S2, Table S5). It is possible that the strong ferromagnetic interaction in **4** is due to the fact that trimers in its structure are joined into infinite chains [-Co(H_2_dapsc)-(2H_2_O)-Co(H_2_dapsc)-Cr(CN)_6_-]*_n_* through the pairs of strong intertrimer hydrogen bonds O-H_H2O_…O_H2daps_ (Figure 7 and Table S4). The metal–metal separations along the formed chain are rather uniform: the Co-Cr distance inside the trimer is 5.128 Å, whereas the Co-Co distance between the trimers is only slightly longer, 5.214 Å. The drop of μ_eff_ to 1.8 K is not as sharp and deep as in the case of complex **2** (Figure 8b,d). This is probably due to the weaker interchain AF interactions in the Co-Co pairs coupled via π-π stacking of the H_2_dapsc ligands in complex **4** (Figure 7) compared to such interactions in infinite Co-Co chains of π-stacked complexes in **2** (similar to the Mn-Mn chains in **1** shown in Figure 3).

#### 2.2.2. Dynamic (ac) Magnetic Properties

In order to examine possible SCM or SMM properties of the complexes **1**, **2** and **3**, **4**, respectively, the in-phase χ*’* and out-of-phase χ″ components of ac magnetic susceptibility were measured in the frequency range of 0.1 Hz–1000 Hz at temperatures 1.8 K–5.0 K in a zero and in non-zero magnetic field. Chain (**2**) and trimeric (**4**) complexes, in which the Co(II) and Cr(III) metal centers are linked by cyanide bridges, did not show a frequency dependence of χ″ in zero and non-zero static magnetic fields, thus precluding the single chain or single molecule magnetic behavior for **2** and **4**, respectively (Figures S5–S7). This result agrees with the statement made in [23] that seven-coordinated Co(II) PBP complexes with planar anisotropy are not suitable as building blocks for the creation of exchange-coupled polynuclear ensembles demonstrating slow magnetization relaxation, in contrast, for example, to PBP complexes of Fe(II) and Ni(II) with Ising anisotropy.

**Figure 10:**
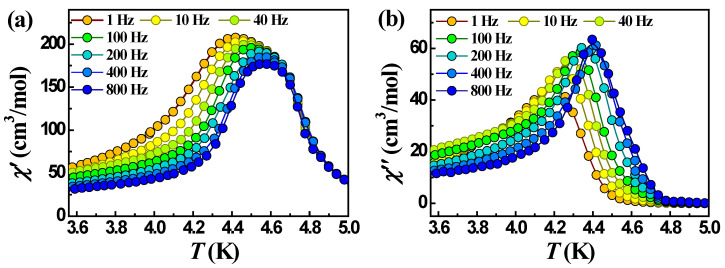
Temperature dependencies of χ*’* (**a**) and χ″ (**b**) at different frequencies for complex **1** in a zero dc field.

Unlike **2** and **4**, complexes **1** and **3** which contain Mn(II) instead of Co(II) exhibit frequency-dependent signals χ*’* and χ″. The plots of temperature and frequency dependencies of χ*’* and χ″ for **1** in a zero dc field are shown in Figure 10 and Figure S8. Below 4.7 K, an out-of-phase signal reveals a frequency-dependent maximum that indicates a slow relaxation of magnetization. However, the relative change of the temperature of the χ″ maximum depending on the frequency of the oscillating field, expressed by the so-called Mydosh parameter ϕ = (ΔTp/Tp)/Δ(logf) [69], turned out to be 0.023, which is in the range of values characteristic of spin glass (0.004 > ϕ < 0.08) and not superparamagnets for which the Mydosh parameter is usually an order of magnitude larger (>0.1) [70,71]. The relaxation time (τ) was fitted to the Arrhenius equation, τ = τ_0_exp(*U*_eff_/k_B_T), where τ = 1/2πν, to allow the estimation of a pre-exponential factor, τ_0_ = 6.5·10^−55^ s, and the effective barrier to the relaxation of magnetization, *U*_eff_ = 512 K (Figure S9). These values are outside the normal range for typical SCMs (τ_0_ usually >10^−13^ s) and indicate that **1** shows typical spin-glass behavior. Similar behavior was found in other 1D chain complexes [72,73,74,75,76]. Antiferromagnetic interactions between different structural units can lead to randomness (disorder, defects) and frustration, which are responsible for the spin-glass system [73,74,76,77]. In **1**, the antiferromagnetic interaction between the chains, transferred through π-π stacking and hydrogen bonds, disrupts the ferrimagnetic ordering in the chains and leads to the disorder of the electron spin system, which causes the spin-glass behavior of complex **1**. Moreover, the loss of the coordination solvent (EtOH), which is involved in the formation of hydrogen bonds in **1**, may contribute to the formation of the spin-glass state, as was observed in the works [73,77].

**Figure 11:**
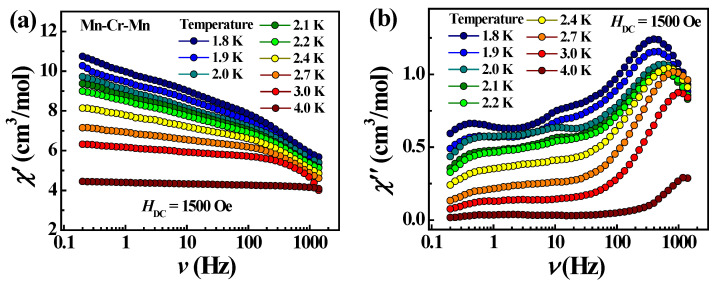
Frequency dependencies of χ*’* (**a**) and χ″ (**b**) at different temperatures for complex **3** in 1500 Oe dc field.

**Figure 12:**
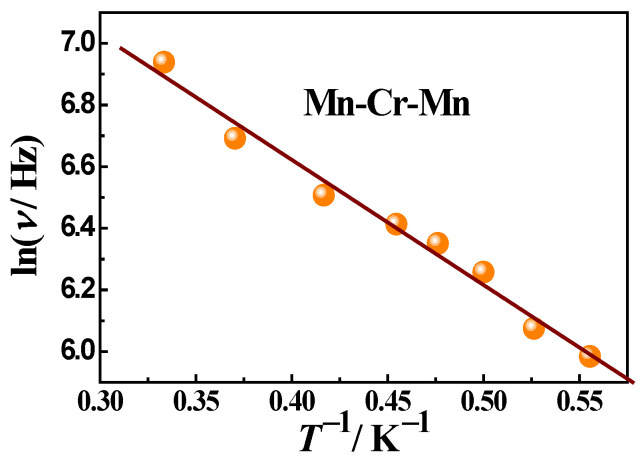
The dependence of lnτ vs. 1/T for **3** in 1500 Oe dc field and its approximation using the Arrhenius formula (solid line). The points were obtained from the frequency dependencies χ″(ν) at different temperatures (Figure 11, main text). Approximation parameters: activation energy *U*_eff_ = 4.1 K, a pre-exponential factor τ_0_ = 4.2·10^−6^ s.

In the case of trimeric complex **3**, the studies of ac susceptibility showed frequency-dependent signals of χ″ when applying a dc field of 1500 Oe (Figure 11), indicating a slow relaxation of the magnetization. The pronounced χ″ maxima appear one after another in the frequency range above 100 Hz and shift towards higher frequencies with increasing temperature, which is typical for SMMs. The Mydosh parameter is 0.9. The temperature dependence of the χ″ peak frequency follows an Arrhenius law with an effective energy barrier *U*_eff_ = 4.1 K and a pre-exponential factor τ_0_ being 4.2·10^−6^ s, suggesting a thermally activated relaxation (Figure 12). Thus, complex **3** is a field-induced SMM.

## 3. Materials and Methods

All chemicals and solvents were reagent grade and used without further purification. The starting compounds [Mn(H_2_dapsc)Cl_2_]·H_2_O [78] and [Co(H_2_dapsc)Cl(H_2_O)]Cl·2H_2_O [31] were prepared according to the literature procedures. The (PPh_4_)_3_[Cr(CN)_6_]·2H_2_O was synthesized by metathesis in water using K_3_[Cr(CN)_6_] and PPh_4_Cl.

The C, H, N, O elemental analyses were carried out with a Vario Micro Cube analyzing device. The infrared spectra were measured on solid samples using a VERTEX 70v (Bruker) spectrometer in the range of 4000–500 cm^−1^. The thermogravimetric analysis (TGA) was performed in an argon atmosphere with a heating rate of 5.0 °C min^−1^ using a NETZSCH STA 409 C Luxx thermal analyzer. X-ray powder diffraction spectra were recorded using a Siemens D500 powder diffractometer with a linear detector at room temperature (Cu*K*α1-radiation, λ = 1.5406 Å, step = 0.02°, single crystal sample holder). The dc and ac magnetic susceptibility of powder samples of complexes **1**–**4** were measured using a Quantum Design MPMS-5 SQUID magnetometer. The experimental data were corrected for the sample holder and for the diamagnetic contribution calculated from Pascal constants.

### 3.1. Synthesis

#### 3.1.1. {[Mn(H_2_dapsc)]Cr^III^(CN)_6_K(H_2_O)_2.5_(EtOH)_0.5_}*_n_*·1.2*n*(H_2_O) (**1**)

The yellow crystals of **1** were obtained by slow diffusion of K_3_[Cr(CN)_6_] solution (30 mg; 0.092 mmol in 3 mL H_2_O) through frit with a pore diameter of 10–20 microns into [Mn(H_2_dapsc)Cl_2_]·H_2_O solution (39 mg; 0.092 mmol) in 10 mL of an ethanol-water (2:1) mixture for two weeks at 8–10 °C. The resulting crystalline precipitate was filtered, washed with ethanol and dried in vacuum. After drying in vacuum, the crystals lost 0.5 molecule of C_2_H_5_OH. Yield: 47 mg (80%). Anal. calc. (%) for C_17_H_22.4_N_13_O_5.7_CrKMn: C, 31.60; H, 3.49; N, 28.18; O, 14.11. Found (%): C, 31.2; H, 3.5; N, 27.9; O, 14.2. This stoichiometry corresponds to the dried sample of **1** as confirmed by TGA analysis (Figure S10). Characteristic IR data (cm^−1^): ν(C≡N) 2152, 2130; ν(C=N) 1663 (imine); ν(Cr-C) 458 (Figure S11).

The thermogram of the dried crystalline sample (Figure S10) demonstrates a mass loss of 7.72% in the temperature range 50–150 °C with an endothermic peak at 111.6 °C which corresponds to the loss of 2.8 molecules H_2_O. In the mass spectrum recorded in the gas phase, the peaks are observed at *m*/*z* = 18 and *m*/*z* = 17 from H_2_O molecules, while peaks from ethanol molecules are not observed. The decomposition of the complex starts above 200 °C with DSC peak at 280.9 °C and is accompanied by the release of CN- (*m*/*z* = 26), OH- (*m*/*z* = 17) and CH_3_^−^ (*m*/*z* = 15) fragments. 

#### 3.1.2. {[Co(H_2_dapsc)]Cr^III^(CN)_6_K(H_2_O)_2.5_(EtOH)_0.5_}*_n_*·1.2*n*(H_2_O) (**2**)

The light orange crystals of **2** were obtained at room temperature by slow diffusion of the starting reagents: [Co(H_2_dapsc)ClH_2_O]Cl·2H_2_O (46.1 mg; 0.1 mmol) in the mixture of 5 mL ethanol and 2 mL water and K_3_[Cr(CN)_6_] (32.5 mg; 0.1 mmol) in 5 mL H_2_O into 10 mL of an ethanol-water (2:1) medium. After 5–7 days, the resulting crystals were filtered, washed with ethanol and dried in vacuum. Yield: 35 mg (~54%). Elemental analysis showed that upon drying the crystals lost 0.5 molecule of C_2_H_5_OH, which was additionally confirmed by the TGA of dried samples (Figure S12). Anal. calc. (%) for C_17_H_22.4_N_13_O_5.7_CoCrK: C, 31.41; H, 3.47; N, 28.01; O, 14.03. Found (%): C, 31.5; H, 3.5; N, 27.9; O, 14.2. Characteristic IR data (cm^−1^): ν(C≡N) 2160, 2129; ν(C=N) 1670 (imine); ν(Cr-C) 456 (Figure S13).

The thermal analysis of complex **2** after drying in vacuum showed that a mass loss begins at ~50 °C with an endothermic peak at 112.7 °C and reaches 9.5% at 150 °C, that corresponds to the loss of 3.5 molecules H_2_O (Figure S12). In the mass spectrum of the gas phase, the peaks are observed at *m*/*z* = 18 and *m*/*z* = 17 from H_2_O molecules. The peaks from ethanol molecules are absent. The second weight-loss step appears above 200 °C with the release of fragments of the decaying complex.

For the single crystal X-ray diffraction analysis, crystals of **1**, **2** were kept in contact with mother liquid to prevent an ethanol loss. After drying, the complexes preserve crystallinity. X-ray powder diffraction pattern of the dried sample of **2** shows a coincidence with the simulated pattern calculated from the single crystal data (Figure S14).

#### 3.1.3. {[Mn(H_2_dapsc)]_2_Cr(CN)_6_(H_2_O)_2_}Cl·H_2_O (**3**)

The crystals of **3** were obtained by slow diffusion of (PPh_4_)_3_[Cr(CN)_6_]·2H_2_O solution (77 mg; 0.06 mmol) in 3 mL methanol into 12 mL methanol-water (3:1) solution of [Mn(H_2_dapsc)Cl_2_]·H_2_O (50 mg; 0.12 mmol) at 8–10 °C in the course of 5-7 days. The resulting crystals were filtered, washed with methanol and dried in vacuum. Yield: 38 mg (65%). Anal. calc. (%) for C_28_H_36_N_20_O_7_ClCrMn_2_: C, 34.95; H, 3.77; N, 29.12; Cl, 3.68. Found (%): C, 35.2; H, 3.8; N, 29.0; Cl, 3.5. Characteristic IR data (cm^−1^): ν(C≡N) 2142, 2131; ν(C=N) 1660 (imine); ν(Cr-C) 448 (Figure S15).

#### 3.1.4. {[Co(H_2_dapsc)]_2_Cr(CN)_6_(H_2_O)_2_}Cl·2EtOH·3H_2_O (**4**)

The crystals of **4** were obtained in an H-shaped tube. One compartment of the H-tube was filled with solution of [Co(H_2_dapsc)ClH_2_O]Cl·2H_2_O (28 mg; 0.06 mmol) in 3 mL of ethanol and 1.5 mL of H_2_O. The second compartment was filled with solution of (PPh_4_)_3_[Cr(CN)_6_]·2H_2_O (38 mg; 0.03 mmol) in 4 mL of ethanol. The tube was filled with ethanol and left in a refrigerator. After three weeks, the resulting orange crystals were filtered, washed with ethanol twice and dried in vacuum. Yield: 14 mg (~45%). Elemental analysis showed that crystals **4** lost the crystallized ethanol. Anal. calc. (%) for C_28_H_40_N_20_O_9_ClCo_2_Cr: C, 33.42; H, 4.01; N, 27.84; Cl, 3.52. Found (%): C, 33.3; H, 4.1; N, 27.5; Cl, 3.5. Characteristic IR data (cm^−1^): ν(C≡N) 2166, 2123; ν(C=N) 1668 (imine); ν(Cr-C) 450 (Figure S16).

### 3.2. Crystal Structure Determination

X-ray single crystal diffraction data were collected at low temperatures on an Oxford Diffraction Gemini-R CCD diffractometer equipped with an Oxford cryostream cooler [λ(Mo*K*_α_) = 0.71073 Å, graphite monochromator, ω-scans]. Single crystals of the complexes **1**–**4** were taken from the mother liquid using a nylon loop with oil and immediately transferred into the cold nitrogen stream of the diffractometer. Data reduction with empirical absorption correction of experimental intensities (Scale3AbsPack program) was made with the CrysAlisPro software [79].

The structures were solved by a direct method and refined by a full-matrix least squares method using SHELX-2016 program [80]. All non-hydrogen atoms were refined anisotropically. The positions of H-atoms were refined in a riding model with isotropic displacement parameters depending on the *U*_eq_ of the connected atom. The hydrogen atoms in water molecules were found from the difference Fourier map. All N-H and O-H bond distances were refined, with additional geometrical restraints (SADI/DFIX) applied in some cases. Acentric structures **1** and **2** were refined as two-component inversion twins, with refined twin fractions of 0.50(1) and 0.48(1), respectively. Main crystal and experimental data for **1**–**4** are listed in Table 1.

## 4. Conclusions

Four heterometallic cyano-bridged complexes combining [M^II^(H_2_dapsc)]^2+^ cations and [Cr(CN)_6_]^3−^ anions were synthesized: {[M(H_2_dapsc)]Cr(CN)_6_K(H_2_O)_2.5_(EtOH)_0.5_}*_n_*·1.2*n*(H_2_O), M = Mn (**1**) and Co (**2**); {[Mn(H_2_dapsc)]_2_Cr(CN)_6_(H_2_O)_2_}Cl·H_2_O (**3**) and {[Co(H_2_dapsc)]_2_Cr(CN)_6_(H_2_O)_2_}Cl·2EtOH·3H_2_O (**4**). The single crystal X-ray diffraction study showed that the complexes contain magnetic M(II) centers in the pentagonal bipyramidal coordination incorporated in the chain (in **1**, **2**) or trimeric (in **3**, **4**) structure by linking through [Cr(CN)_6_] bridging units. The pentadentate H_2_dapsc ligands in all four compounds keep the protons in both hydrazine –NH moieties and retain a neutral state. Structural analysis revealed the important role of intermolecular non-covalent interactions such as hydrogen bonding and π…π stacking in the stabilization of crystal packing. In the chain compounds **1** and **2**, neighbor chains are linked additionally by coordination bonds via pairs of potassium cations and oxygen atoms from water/ethanol molecules. Pairs of compounds **1**, **3** and **2**, **4** containing Mn(II) and Co(II), respectively, differ significantly in their dc and ac magnetic properties. In contrast to **1**, in compound **2**, a field-induced metamagnetic transition with a critical field of 1500 Oe was found. Complexes **1** and **3** exhibit a frequency-dependent ac magnetic susceptibility due to the spin-glass behavior in the chain complex **1** and the field-induced single molecule magnetism in the trimeric complex **3**. At the same time, **2** and **4** did not show a frequency dependence of χ″ signals of ac susceptibility in the zero and non-zero dc field, thus precluding the single chain or single molecule magnetic behavior for **2** and **4**, respectively. This result implies that seven-coordinated Co(II) PBP complexes with planar anisotropy are not suitable as building units for the design of advanced molecular nanomagnets based on the M(4d/5d)–M(3d) exchange-coupled pairs with Ising-type exchange interactions [10,81]. At the same time, Mn(II) PBP complexes with high spin (S = 5/2) can be used for these purposes. 

## Figures and Tables

**Table 1:** Crystal data and structural refinement parameters for the complexes **1**–**4**.

	1	2	3	4
Chemical formula	C_18_H_25.4_CrKMnN_13_O_6.2_	C_18_H_25.4_CoCrKN_13_O_6.2_	C_28_H_36_ClCrMn_2_N_20_O_7_	C_32_H_52_ClCo_2_CrN_20_O_11_
Formula weight	669.15	673.14	962.10	1098.24
Cell setting	monoclinic	monoclinic	monoclinic	triclinic
Space group, *Z*	*P*2_1_, 4	*P*2_1_, 4	*P*2_1_/*c*, 2	$P\bar{1}$, 1
Temperature (K)	120(1)	120(1)	150(1)	150(1)
*a* (Å)	9.3525(2)	9.4213(1)	10.5837(4)	9.7054(10)
*b* (Å)	16.6438(3)	16.5874(2)	15.2401(5)	11.2166(14)
*c* (Å)	18.5601(3)	18.1419(2)	12.3806(4)	11.4125(13)
α (^o^)	90	90	90	71.915(11)
β (^o^)	90.709(1)	90.428(1)	103.326(4)	88.039(9)
γ (^o^)	90	90	90	85.191(9)
Cell volume (Å^3^)	2888.86(8)	2835.05(6)	1943.18(12)	1176.8(2)
ρ (g/cm^3^)	1.539	1.577	1.644	1.550
μ, cm^−1^	10.15	11.74	10.56	10.56
Refls colld/unique	41,136/14,870	32,709/14,858	14,493/5499	9774/5136
*R*_int_	0.0194	0.0280	0.0198	0.0924
θ_max_ (^o^)	29.00	29.00	29.00	26.00
Parameters refined	814	826	312	341
*R*_1_, *wR*_2_ [*I* > 2σ(*I*)]	0.0285, 0.0695	0.0328, 0.0718	0.0292, 0.0671	0.0932, 0.2297
Goodness-of-fit	1.002	1.001	1.002	1.001
CCDC number	2219102	2219103	2219104	2219105

## Data Availability

The data presented in this study are available in the present article and the Supplementary Materials section.

## Supplementary Materials

The following supporting information can be downloaded at: https://www.mdpi.com/article/10.3390/molecules27238518/s1, Cif and CheckCif files; Figure S1: Interchain hydrogen bonds in the (0 1 0) plane of the structure **1**; Figure S2: Temperature dependencies of inverted magnetic susceptibility for **1**–**4**; Figure S3: Temperature dependencies of the magnetization (a) and dc magnetic susceptibility (b) for **2** at different magnetic fields; Figure S4: Magnetic dc field dependencies of real (χ’) and imaginary (χ″) parts of ac magnetic susceptibility at 2.0 K for complex **2**; Figure S5: Frequency dependencies of ac magnetic susceptibility at 2.0, 2.5 and 3.0 K for complex **2** in a zero dc field; Figure S6: Frequency dependencies of ac magnetic susceptibility at 2.0–3.5 K temperature range for complex **2** under 1.5 kOe dc field; Figure S7: Frequency dependencies of ac magnetic susceptibility at 1.8–5.0 K temperature range for complex **4** under 1.5 kOe dc field; Figure S8: Frequency dependencies of χ’ and χ″ at different temperatures for complex **1** in a zero dc field; Figure S9: The dependence of ln(τ) vs. *T*^−1^ for **1** in a zero dc field; Figure S10: TG-DSC curves and mass spectra for complex **1** after drying in vacuum; Figure S11: IR (a) and Raman (b) spectra of the complex **1**; Figure S12: TG-DSC curves and mass spectra for complex **2** after drying in vacuum; Figure S13: IR spectrum of the complex **2**; Figure S14: Powder diffraction pattern for a dried sample of **2**; Figure S15: IR spectrum of the complex **3**; Figure S16: IR spectrum of the complex **4**; Tables S1, S2, S3, S4: Hydrogen bond geometry in **1**, **2**, **3**, **4**, respectively; Table S5: Weiss temperatures in the complexes **1**–**4**.

## Appendix A

**Table A1:** Selected bond lengths (Å) and angles (°) in **1**–**4**.

1			
Mn(1)-O(1)	2.244(2)	Mn(2)-O(3)	2.272(2)
Mn(1)-O(2)	2.255(2)	Mn(2)-O(4)	2.218(2)
Mn(1)-N(5)	2.291(2)	Mn(2)-N(12)	2.274(2)
Mn(1)-N(6)	2.311(2)	Mn(2)-N(13)	2.310(2)
Mn(1)-N(7)	2.306(3)	Mn(2)-N(14)	2.282(3)
Mn(1)-N(41)	2.210(3)	Mn(2)-N(42) ^d^	2.227(3)
Mn(1)-N(51)	2.221(2)	Mn(2)-N(52) ^c^	2.214(3)
Cr(1)-C_CN_	2.063(3)–2.080(3)	Cr(2)-C_CN_	2.070(3)–2.076(3)
O(1)-Mn(1)-O(2)	84.21(7)	O(3)-Mn(2)-O(4)	83.08(7)
O(1)-Mn(1)-N(5)	70.68(8)	O(3)-Mn(2)-N(12)	70.41(8)
O(2)-Mn(1)-N(6)	69.53(8)	O(4)-Mn(2)-N(13)	69.76(8)
N(5)-Mn(1)-N(7)	68.14(9)	N(12)-Mn(2)-N(14)	68.77(9)
N(6)-Mn(1)-N(7)	67.46(8)	N(13)-Mn(2)-N(14)	67.98(9)
N(41)-Mn(1)-N(51)	171.0(1)	N(42)^d^-Mn(2)-N(52) ^c^	170.0(1)
Mn(1)-N(41)-C(41)	152.3(2)	Mn(2)-N(42)^d^-C(42) ^d^	151.8(2)
Mn(1)-N(51)-C(51)	151.4(2)	Mn(2)-N(52)^c^-C(52)^c^	149.6(2)
Cr(1)-C(41)-N(41)	173.3(3)	Cr(1)-C(42)-N(42)	173.1(3)
Cr(2)-C(51)-N(51)	175.2(2)	Cr(2)-C(52)-N(52)	175.9(2)
**2**			
Co(1)-O(1)	2.208(2)	Co(2)-O(3)	2.243(2)
Co(1)-O(2)	2.225(2)	Co(2)-O(4)	2.189(2)
Co(1)-N(5)	2.204(3)	Co(2)-N(12)	2.185(3)
Co(1)-N(6)	2.220(3)	Co(2)-N(13)	2.218(3)
Co(1)-N(7)	2.205(3)	Co(2)-N(14)	2.178(3)
Co(1)-N(41)	2.091(3)	Co(2)-N(42)^d^	2.097(3)
Co(1)-N(51)	2.099(3)	Co(2)-N(52)^c^	2.098(3)
Cr(1)-C_CN_	2.068(3)–2.081(4)	Cr(2)-C_CN_	2.063(4)–2.079(4)
O(1)-Co(1)-O(2)	77.5(1)	O(3)-Co(2)-O(4)	76.4(1)
O(1)-Co(1)-N(5)	71.9(1)	O(3)-Co(2)-N(12)	71.6(1)
O(2)-Co(1)-N(6)	70.8(1)	O(4)-Co(2)-N(13)	71.2(1)
N(5)-Co(1)-N(7)	70.3(1)	N(12)-Co(2)-N(14)	70.8(1)
N(6)-Co(1)-N(7)	69.5(1)	N(13)-Co(2)-N(14)	70.0(1)
N(41)-Co(1)-N(51)	170.2(1)	N(42)^d^-Co(2)-N(52) ^c^	170.6(1)
Co(1)-N(41)-C(41)	158.3(3)	Co(2)-N(42)^d^-C(42) ^d^	160.9(3)
Co(1)-N(51)-C(51)	155.2(3)	Co(2)-N(52)^c^-C(52)^c^	154.9(3)
Cr(1)-C(41)-N(41)	171.1(3)	Cr(1)-C(42)-N(42)	170.4(3)
Cr(2)-C(51)-N(51)	173.4(3)	Cr(2)-C(52)-N(52)	173.3(3)
**3**		**4**	
Mn(1)-O(1)	2.205(1)	Co(1)-O(1)	2.157(4)
Mn(1)-O(2)	2.272(1)	Co(1)-O(2)	2.168(3)
Mn(1)-N(5)	2.301(1)	Co(1)-N(5)	2.182(4)
Mn(1)-N(6)	2.285(1)	Co(1)-N(6)	2.181(4)
Mn(1)-N(7)	2.323(1)	Co(1)-N(7)	2.171(4)
Mn(1)-N(8)	2.226(1)	Co(1)-N(8)	2.106(4)
Mn(1)-O(3)	2.265(1)	Co(1)-O(3)	2.164(3)
Cr(1)-C_CN_	2.069(1)–2.087(1)	Cr(1)-C_CN_	2.058(5)–2.072(6)
O(1)-Mn(1)-O(2)	85.12(4)	O(1)-Co(1)-O(2)	74.8(1)
O(1)-Mn(1)-N(5)	70.34(4)	O(1)-Co(1)-N(5)	71.6(1)
O(2)-Mn(1)-N(6)	69.26(4)	O(2)-Co(1)-N(6)	72.3(1)
N(5)-Mn(1)-N(7)	67.21(4)	N(5)-Co(1)-N(7)	70.9(2)
N(6)-Mn(1)-N(7)	67.75(4)	N(6)-Co(1)-N(7)	70.5(1)
N(8)-Mn(1)-O(3)	165.80(4)	N(8)-Co(1)-O(3)	176.4(1)
Mn(1)-N(8)-C(12)	164.7(1)	Co(1)-N(8)-C(12)	151.3(4)
Cr(1)-C(12)-N(8)	174.1(1)	Cr(1)-C(12)-N(8)	176.9(4)

Symmetry codes: ^c^ (−*x*, *y* + 0.5, −*z*), ^d^ (1 − *x*, *y* + 0.5, 1 − *z*).
