# Supplementary material for: [MII(H2dapsc)]-[Cr(CN)6] (M = Mn, Co) Chain and Trimer Complexes: Synthesis, Crystal Structure, Non-Covalent Interactions and Magnetic Properties
Source: Molecules. 2022 Dec 3;27(23):8518. doi: 10.3390/molecules27238518 (PMC9737345; doi:10.3390/molecules27238518)
Supplement: Supplementary file 1 [file molecules-27-08518-s001.zip › molecules-2067096-supplementary.pdf]

Supplementary Information

**Figure S1**	Interchain hydrogen bonds in the (0 1 0) plane of the structure **1**	p. S2
**Table S1**	Hydrogen bond geometry in **1**.	p. S2
**Table S2**	Hydrogen bond geometry in **2**.	p. S3
**Table S3**	Hydrogen bond geometry in **3**.	p. S4
**Table S4**	Hydrogen bond geometry in **4**.	p. S4
**Figure S2**	Temperature dependences of inverted magnetic susceptibility for **1-4**.	p. S5
**Table S5**	Weiss temperatures in the complexes **1-4**.	p. S5
**Figure S3**	Temperature dependences of the magnetization (a) and dc magnetic susceptibility (b) for **2** at different magnetic fields.	p. S5
**Figure S4**	Magnetic dc field dependences of real (χ') and imaginary (χ'') parts of ac magnetic susceptibility at T = 2.0 K for complex **2**.	p. S6
**Figure S5**	Frequency dependencies of ac magnetic susceptibility at 2.0, 2.5 and 3.0 K for complex **2** in a zero dc field.	p. S6
**Figure S6**	Frequency dependencies of ac magnetic susceptibility at 2.0-3.5 K temperature range for complex **2** under 1.5 kOe dc field.	p. S7
**Figure S7**	Frequency dependencies of ac magnetic susceptibility at 1.8-5.0 K temperature range for complex **4** under 1.5 kOe dc field.	p. S7
**Figure S8**	Frequency dependencies of χ' and χ'' at different temperatures for complex **1** in a zero dc field.	p. S8
**Figure S9**	The dependence of ln(τ) *vs* T^{-1} for **1** in a zero dc field.	p. S8
**Figure S10**	TG-DSC curves and mass spectra for complex **1** after drying in vacuum.	p. S9
**Figure S11**	IR (a) and Raman (b) spectra of the complex **1**.	p. S10
**Figure S12**	TG-DSC curves and mass spectra for complex **2** after drying in vacuum.	p. S11
**Figure S13**	IR spectrum of the complex **2**.	p. S11
**Figure S14**	Powder diffraction pattern for a dried sample of **2**.	p. S12
**Figure S15**	IR spectrum of the complex **3**.	p. S13
**Figure S16**	IR spectrum of the complex **4**.	p. S13

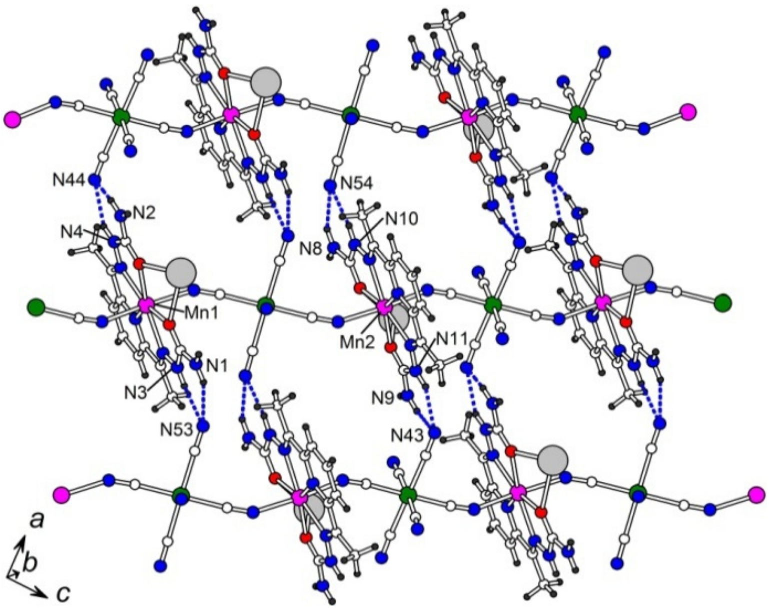

Figure S1. Interchain hydrogen bonds (shown by blue dashed lines) in the (0 1 0) plane of the structure **1** (water and ethanol molecules are omitted for clarity). Hydrogen bonds are shown by blue dashed lines (see Tables S1 and S2 for hydrogen bond geometry in **1** and **2**, respectively).

Table S1. Hydrogen bond geometry in **1**.

Donor--H..Acceptor	[symmetry]	D-H, Å	H...A, Å	D...A, Å	D-H...A, °	
N1 --H1B ..N53	[x+1,y,z]	0.83(3)	2.27(3)	3.052(4)	157(3)	Fig. S1
N3 --H3 ..N53	[x+1,y,z]	0.83(2)	2.19(3)	2.945(4)	152(3)	Fig. S1
N2 --H2B ..N44	[x+1,y,z]	0.83(3)	2.50(3)	3.251(4)	150(3)	Fig. S1
N4 --H4 ..N44	[x-1,y,z]	0.84(3)	2.16(3)	2.932(4)	155(3)	Fig. S1
N8 --H8B ..N54	[1-x,y+0.5,-z]	0.83(3)	2.28(3)	3.036(4)	152(3)	Fig. S1
N10 --H10 ..N54	[1-x,y+0.5,-z]	0.83(3)	2.18(3)	2.914(4)	148(3)	Fig. S1
N9 --H9B ..N43	[-x,y+0.5,1-z]	0.83(3)	2.53(3)	3.239(4)	143(3)	Fig. S1
N11 --H11 ..N43	[-x,y+0.5,1-z]	0.84(3)	2.10(3)	2.903(4)	162(3)	Fig. S1
N1 --H1A ..O5W	[x,y,z]	0.83(3)	2.09(3)	2.904(4)	169(3)	Fig. 2
N2 --H2A ..O3W	[x,y,z]	0.83(2)	2.17(2)	2.994(4)	172(3)	Fig. 2
N8 --H8A ..O7W	[x,y,z]	0.84(3)	2.12(3)	2.938(4)	166(3)	
N9 --H9A ..O4WA	[x,y,z]	0.83(2)	2.08(2)	2.904(4)	169(3)	Fig. 2
N9 --H9A ..O4WB	[x,y,z]	0.83(2)	2.16(2)	2.899(4)	148(3)	
O2W --H2WB ..N45	[1-x,y+0.5,1-z]	0.82	2.46	3.172(4)	146	Fig. 2
O3W --H3WB ..N45	[1-x,y+0.5,1-z]	0.80(3)	2.27(3)	3.044(3)	163(3)	Fig. 2
O6W --H6WB ..N45	[1-x,y+0.5,1-z]	0.82(4)	2.14(4)	2.924(4)	161(3)	Fig. 2
O3W --H3WA ..N46	[x-1,y,z]	0.82(3)	2.06(3)	2.823(3)	156(3)	
O4WA--H4WA ..N46	[x-1,y,z]	0.80(4)	2.29(5)	3.039(4)	156(4)	
O5W --H5WB ..N56	[x,y,z]	0.83	2.19	2.833(4)	134	Fig. 2
O7W --H7WB ..N56	[x,y,z]	0.82(4)	2.50(4)	3.121(5)	134(3)	
O7W --H7WA ..N55	[-x,y+0.5,-z]	0.81(4)	1.97(4)	2.777(4)	175(3)	
O8W --H8WA ..N55	[-x,y+0.5,-z]	0.81	2.39	3.194(5)	169	
O8W --H8WB ..O3	[x,y,z]	0.82	2.37	2.971(6)	131	

O8W --H8WB ..O4	[x,y,z]	0.82	2.48	3.095(5)	132
O9W --H9WA ..N54	[-x,y+0.5,-z]	0.83	2.56	3.369(6)	168
O1W --H1W ..O7W	[x,y,z]	0.81(3)	1.94(3)	2.725(3)	163(5)
O2W --H2WA ..O9W	[x+1,y,z]	0.83	2.11	2.910(6)	161
O4WA--H4WB ..O6W	[x-1,y,z]	0.82(4)	2.05(4)	2.826(4)	156(5)
O5W --H5WA ..O8W	[x+1,y,z]	0.83	2.29	3.110(6)	169
O9W --H9WB ..O8W	[x,y,z]	0.82	2.01	2.756(7)	151

Table S2. Hydrogen bond geometry in **2**.

Donor--H..Acceptor	[symmetry]	D-H, Å	H...A, Å	D...A, Å	D-H...A, º
N1 --H1B ..N53	[x+1,y,z]	0.82(4)	2.43(4)	3.137(5)	146(4)
N3 --H3 ..N53	[x+1,y,z]	0.81(3)	2.16(4)	2.905(4)	153(4)
N2 --H2B ..N44	[x+1,y,z]	0.81(4)	2.55(3)	3.269(5)	148(3)
N4 --H4 ..N44	[x+1,y,z]	0.81(3)	2.15(4)	2.925(4)	161(3)
N8 --H8B ..N54	[1-x,y+0.5,-z]	0.81(3)	2.40(4)	3.100(4)	145(4)
N10 --H10 ..N54	[1-x,y+0.5,-z]	0.80(3)	2.12(3)	2.876(4)	157(5)
N9 --H9B ..N43	[-x,y+0.5,1-z]	0.81(4)	2.60(5)	3.272(5)	142(4)
N11 --H11 ..N43	[-x,y+0.5,1-z]	0.82(3)	2.12(3)	2.885(4)	157(3)
N1 --H1A ..O5W	[x,y,z]	0.81(3)	2.19(4)	2.930(6)	152(4)
N2 --H2A ..O3W	[x,y,z]	0.81(2)	2.15(2)	2.945(4)	168(4)
N8 --H8A ..O7W	[x,y,z]	0.82(3)	2.16(3)	2.965(5)	168(3)
N9 --H9A ..O4WA	[x,y,z]	0.81(2)	2.11(3)	2.889(6)	161(5)
N9 --H9A ..O4WB	[x,y,z]	0.81(2)	2.06(3)	2.811(6)	154(5)
O2W --H2WB ..N45	[1-x,y+0.5,1-z]	0.88(4)	2.34(3)	3.129(5)	150(5)
O3W --H3WB ..N45	[1-x,y+0.5,1-z]	0.82(4)	2.25(4)	3.014(4)	156(4)
O6W --H6WB ..N45	[1-x,y+0.5,1-z]	0.80(4)	2.34(5)	2.970(5)	137(4)
O3W --H3WA ..N46	[x-1,y,z]	0.83(3)	2.05(4)	2.810(5)	151(4)
O4WA--H4WA ..N46	[x-1,y,z]	0.82(6)	2.25(6)	3.041(6)	164(7)
O5W --H5WB ..N56	[x,y,z]	0.83	2.19	2.874(6)	140
O7W --H7WB ..N56	[x,y,z]	0.83(4)	2.53(5)	3.177(6)	136(4)
O7W --H7WA ..N55	[-x,y+0.5,-z]	0.82(4)	1.94(5)	2.755(5)	169(4)
O8W --H8WA ..N55	[-x,y+0.5,-z]	0.79(6)	2.36(5)	3.095(5)	156(6)
O8W --H8WB ..O3	[x,y,z]	0.78(6)	2.40(6)	2.966(6)	130(5)
O8W --H8WB ..O4	[x,y,z]	0.78(6)	2.23(5)	2.916(5)	147(5)
O9W --H9WA ..N54	[-x,y+0.5,-z]	0.82	2.46	3.246(6)	161
O1W --H1W ..O7W	[x,y,z]	0.92(5)	1.87(5)	2.716(4)	153(4)
O2W --H2WA ..O9W	[x+1,y,z]	0.87(4)	2.00(5)	2.862(7)	173(4)
O4WA--H4WB ..O6W	[x-1,y,z]	0.82(6)	2.35(7)	2.810(6)	117(6)
O5W --H5WA ..O8W	[x+1,y,z]	0.82	2.10	2.921(6)	172
O9W --H9WB ..O8W	[x,y,z]	0.84	1.92	2.702(8)	156

Table S3. Hydrogen bond geometry in **3**.

Donor--H..Acceptor			[symmetry]	D-H, Å	H...A, Å	D...A, Å	D-H...A, °	
N1	--H1A	..O1	[-x, -y, -z]	0.85(2)	2.20(2)	2.987(2)	154(2)	Fig. 5
N1	--H1A	..O2	[-x, -y, -z]	0.85(2)	2.43(2)	2.910(2)	117(2)	
N1	--H1B	..Cl1	[-x,y-0.5,0.5-z]	0.85(2)	2.37(2)	3.180(3)	159(2)	
N2	--H2A	..Cl1	[x,0.5-y,z-0.5]	0.83(2)	2.83(2)	3.660(2)	174(2)	
N3	--H3	..Cl1	[-x,y-0.5,0.5-z]	0.82(2)	2.51(2)	3.228(2)	148(2)	
N3	--H3	..O4	[-x,y-0.5,0.5-z]	0.82(2)	2.34(2)	3.109(5)	158(2)	
N4	--H4	..N10	[x, 0.5-y, z+0.5]	0.83(2)	2.08(2)	2.895(2)	167(2)	
O3	--H3WA	..N9	[x-1,y,z]	0.84(2)	2.04(2)	2.869(2)	173(2)	Fig. 5
O3	--H3WB	..Cl1	[x,y,z]	0.83(2)	2.31(2)	3.194(2)	158(2)	Fig. 5
O3	--H3WC	..Cl1	[-x,-y,1-z]	0.83(2)	2.33(3)	3.110(2)	160(3)	Fig. 5
O4	--H4A	..O3	[x,y,z]	0.83(2)	2.45(4)	3.141(5)	140(3)	Fig. 5
O4	--H4B	..O3	[-x,-y,1-z]	0.83(2)	1.98(3)	2.806(5)	177(3)	Fig. 5

Table S4. Hydrogen bond geometry in **4**.

Donor--H..Acceptor			[symmetry]	D-H, Å	H...A, Å	D...A, Å	D-H...A, °	
N1	--H1A	..O4	[-x,1-y,-z]	0.81(3)	2.16(2)	2.933(6)	160(1)	
N1	--H1B	..N9	[-x,-y,-z]	0.81(3)	2.51(2)	3.171(6)	140(1)	Fig. 7
N3	--H3	..N9	[-x,-y,-z]	0.81(3)	2.15(2)	2.890(6)	152(1)	Fig. 7
N2	--H2A	..Cl1	[-x,1-y,-z]	0.81(3)	2.26(2)	3.065(6)	175(2)	
N2	--H2A	..O6	[-x,1-y,-z]	0.81(3)	2.09(2)	2.903(6)	173(2)	
N2	--H2B	..N8	[1-x,1-y,-z]	0.81(3)	2.59(2)	3.278(6)	144(1)	Fig. 7
N4	--H4	..N8	[1-x,1-y,-z]	0.80(3)	2.67(2)	3.297(6)	137(1)	Fig. 7
O3	--H3A1	..Cl1	[x,y,z]	0.87(3)	2.14(1)	3.009(5)	170(1)	
O3	--H3B1	..O1	[-x,1-y,-z]	0.88(3)	1.99(2)	2.868(5)	177(1)	Fig. 7
O3	--H3A2	..O4	[x,y,z]	0.88(3)	1.91(2)	2.752(6)	160(1)	
O3	--H3B2	..O2	[-x,1-y,-z]	0.88(3)	2.12(2)	2.955(5)	159(1)	Fig. 7
O4	--H4A	..O3	[x,y,z]	0.88(3)	1.89(2)	2.752(5)	165(1)	
O4	--H4B	..O5	[x,y,z]	0.88(3)	1.86(2)	2.711(6)	163(1)	
O5	--H5A	..N10	[1-x,1-y,-z]	0.87(3)	1.91(2)	2.782(6)	175(1)	
O5	--H5B1	..O4	[x,y,z]	0.88(3)	1.91(2)	2.711(6)	152(1)	
O5	--H5B2	..Cl1	[-x,1-y,1-z]	0.87(3)	2.01(2)	2.858(7)	164(1)	
O6	--H6A	..O3	[x,y,z]	0.88(3)	2.11(2)	2.983(5)	171(1)	
O6	--H6B	..O5	[-x,1-y,1-z]	0.88(3)	2.17(2)	3.032(6)	167(1)	

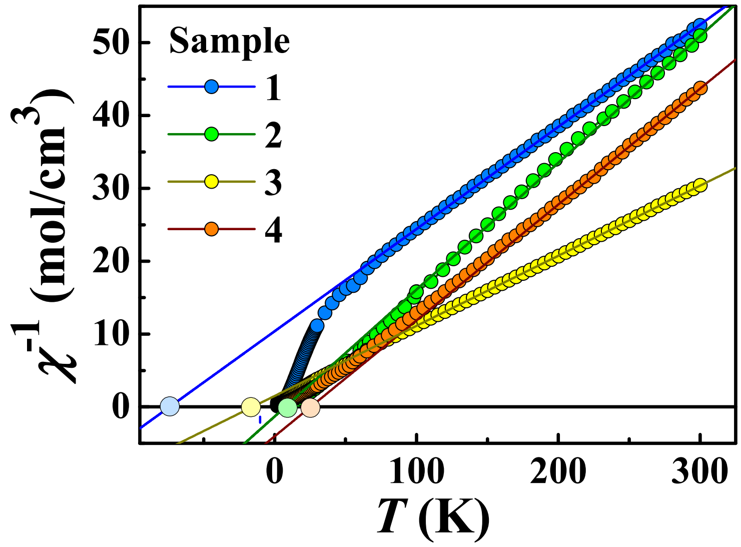

Figure S2. Temperature dependences of inverted magnetic susceptibility for the complexes **1** - **4**. Solid lines show approximations by Curie-Weiss law.

Table S5. Weiss temperatures in the complexes **1**, **2**, **3** and **4**.

Complexes	T_{Weiss}
**1**	-75 K
**2**	+7.0 K
**3**	-15.6 K
**4**	+27 K

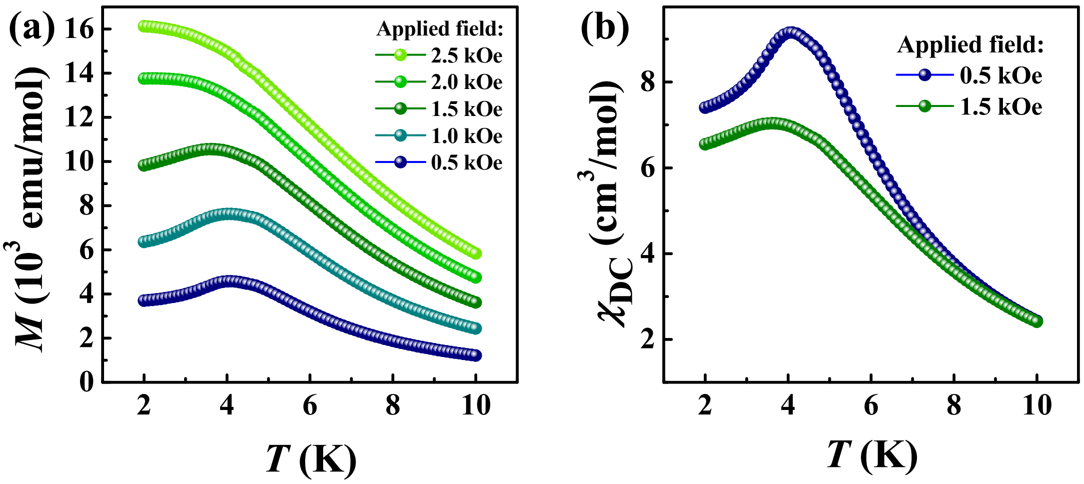

Figure S3. Temperature dependences of the magnetization M (a) and DC magnetic susceptibility (b) for **2** recorded at different magnetic fields.

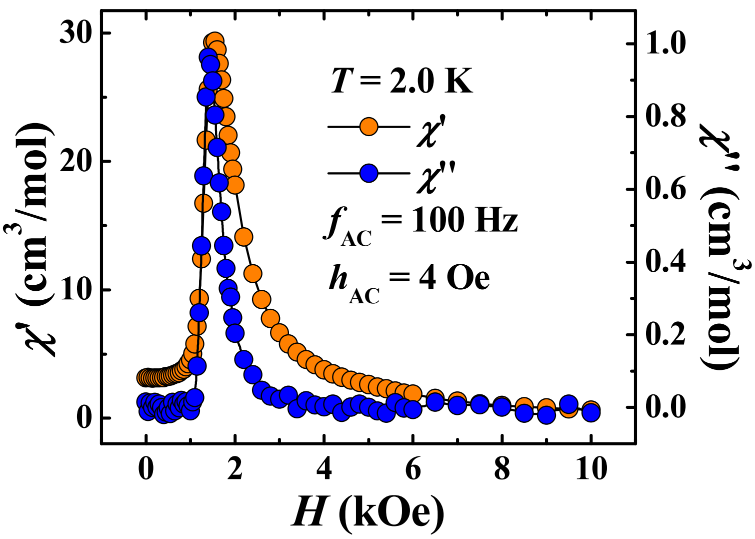

Figure S4. Magnetic dc field dependences of real (χ') and imaginary (χ″) parts of ac magnetic susceptibility at $T = 2.0$ K for complex **2**. AC field amplitude and frequency are h_{AC} = 4 Oe and f_{AC} = 100 Hz, respectively. The maximum of χ' at the dc field H_{MAX} = 1.5 kOe corresponds to metamagnetic transition. The response of χ″ to the transition can be detected, but the χ″ value is only of 3 percent of χ'.

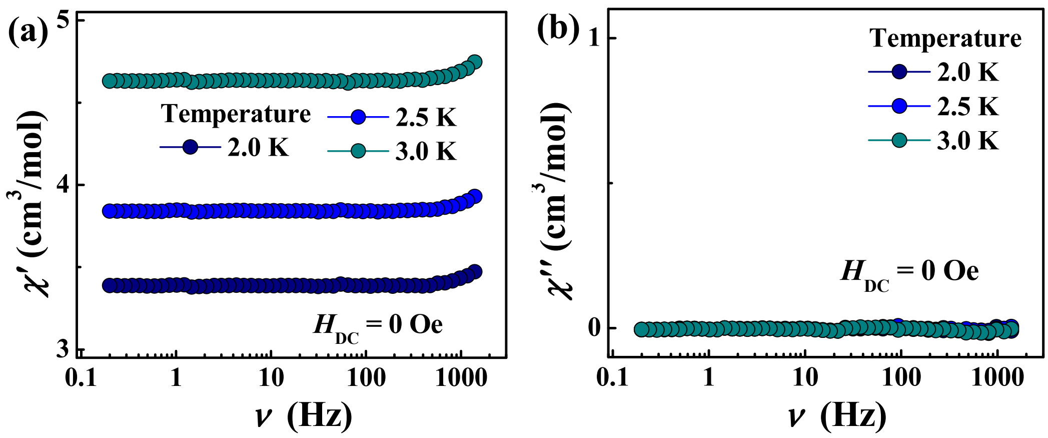

Figure S5. Frequency dependencies of ac magnetic susceptibility at 2.0 K, 2.5 K and 3.0 K for complex **2** in a zero dc field. The ac-field amplitude was h_{AC} = 4 Oe.

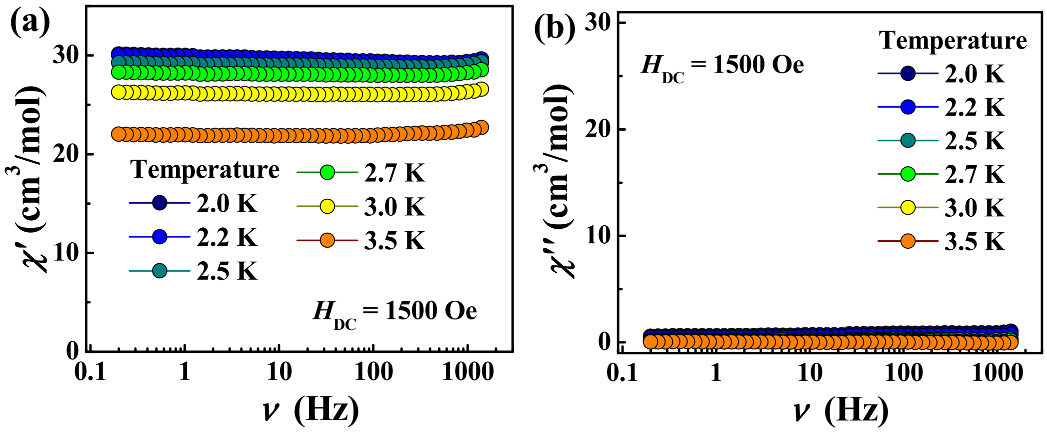

Figure S6. Frequency dependencies of ac magnetic susceptibility at 2.0 K – 3.5 K temperature range for complex **2**, recorded under 1.5 kOe dc field.

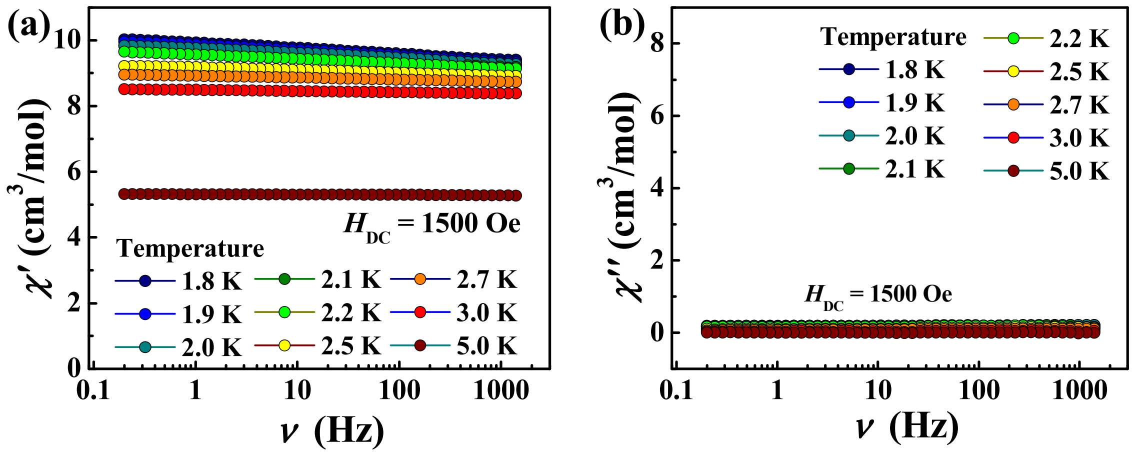

Figure S7. Frequency dependencies of ac magnetic susceptibility at 1.8 K – 5.0 K temperature range for complex **4**, recorded under 1.5 kOe dc field.

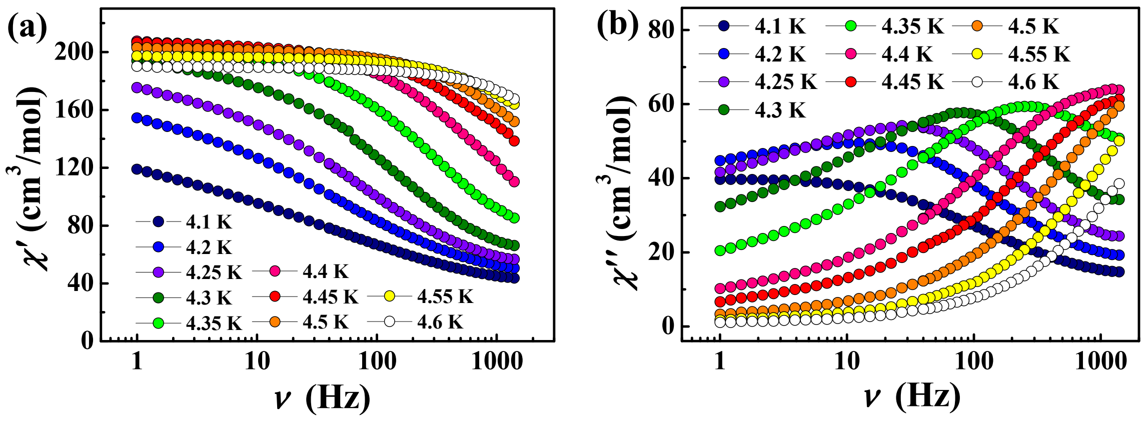

Figure S8. Frequency dependencies of χ' and χ″ at different temperatures for complex **1** in a zero dc field.

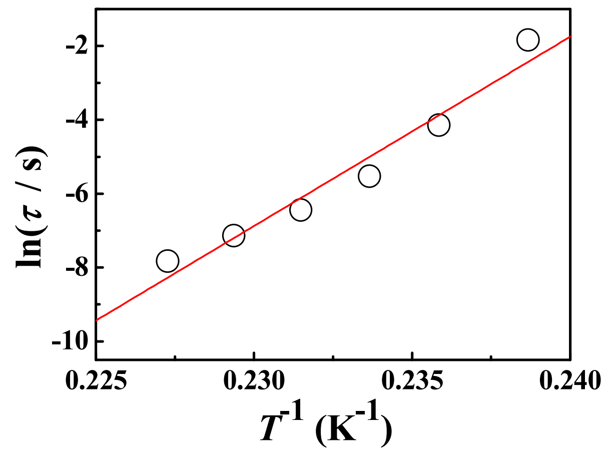

Figure S9. The dependence of ln(τ) *vs* T^{-1} for **1** in a zero dc field and its approximation by the Arrhenius formula (solid line). The points were obtained from the temperature dependences χ″(T) at different frequencies (Figure 10, main text). Approximation parameters: activation energy U_{eff} = 512 K, pre exponential factor $\tau_0 = 6.5\cdot10^{-55}$ s.

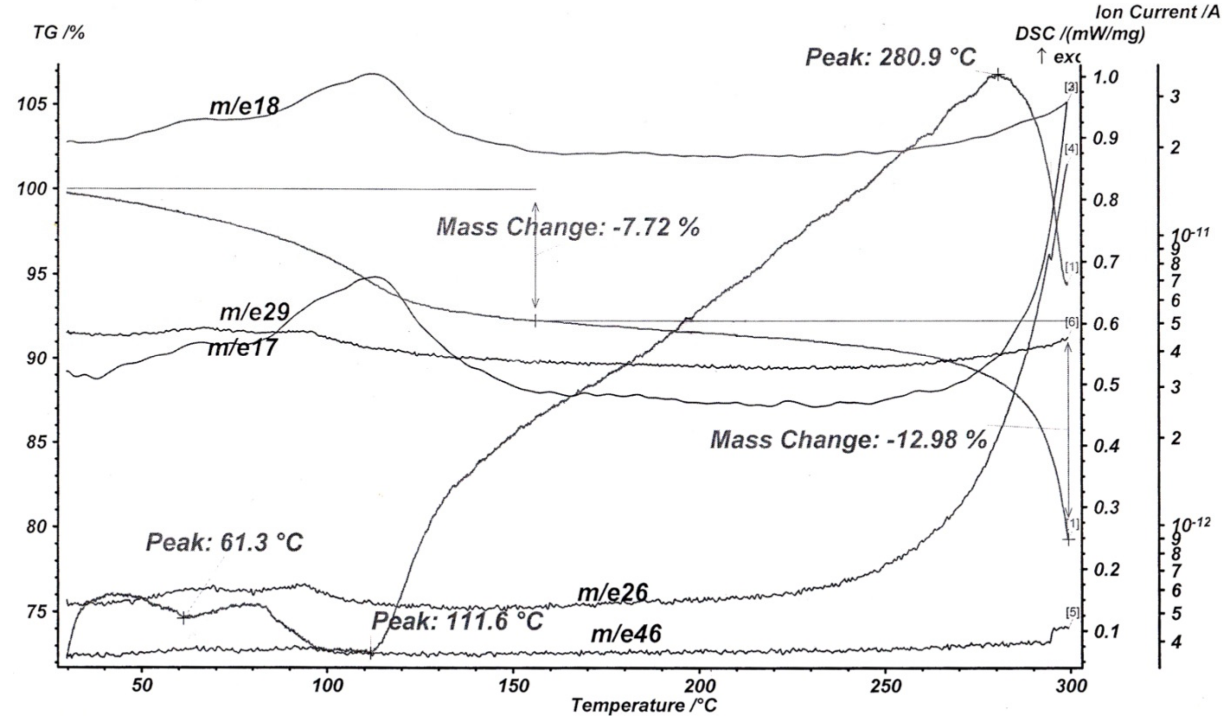

Figure S10. TG-DSC curves and mass spectra for complex **1** after drying in vacuum.

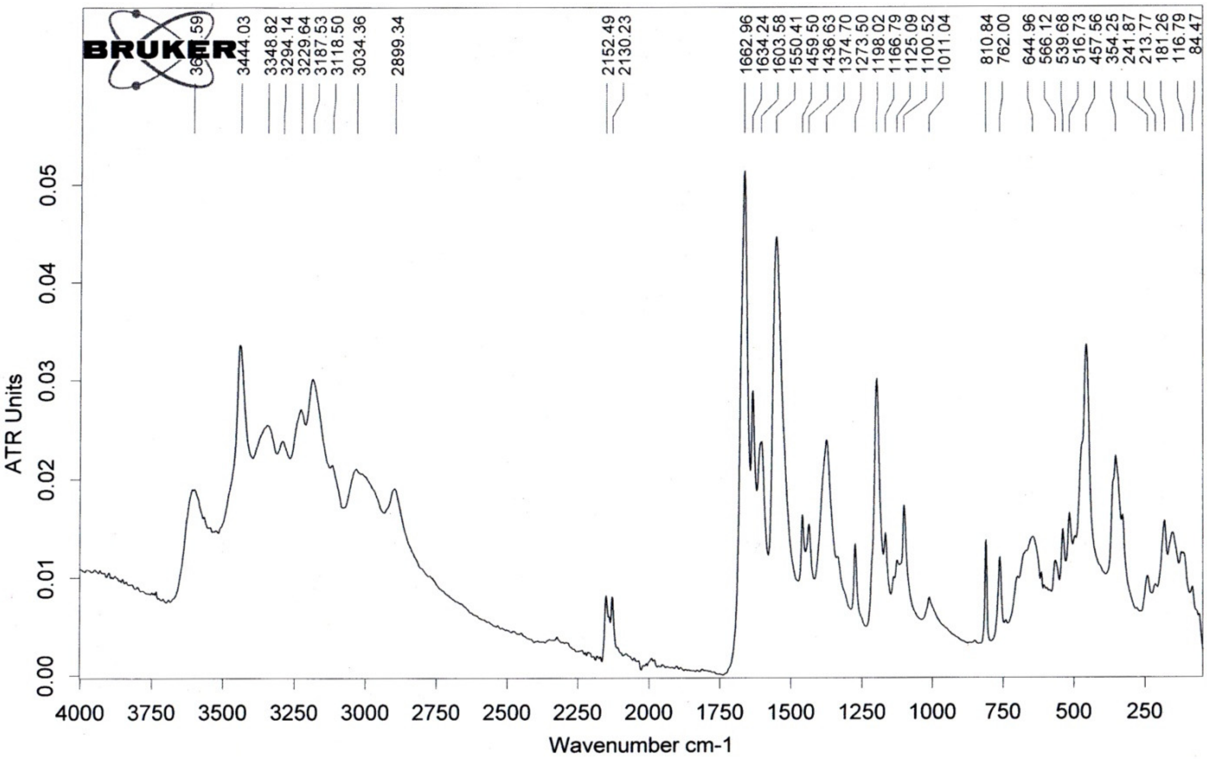

(a)

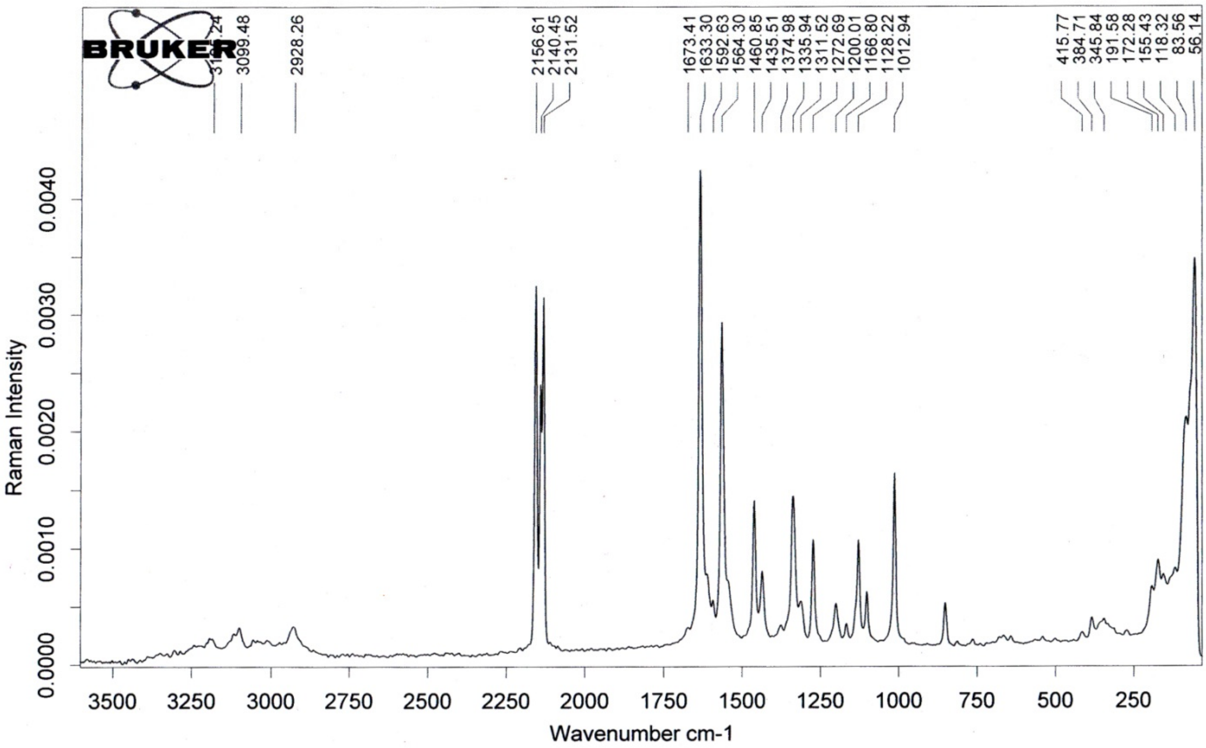

(b)

Figure S11. IR (a) and Raman (b) spectra of the complex **1.**

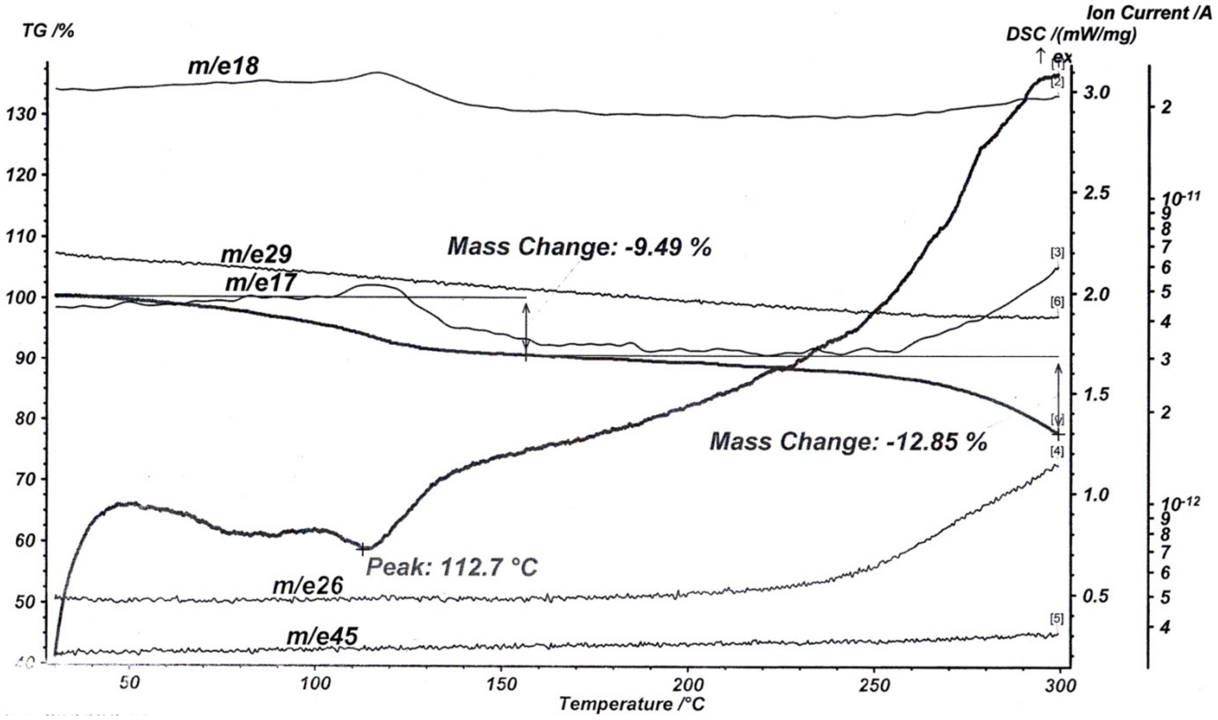

Figure S12. TG-DSC curves and mass spectra for complex **2** after drying in vacuum.

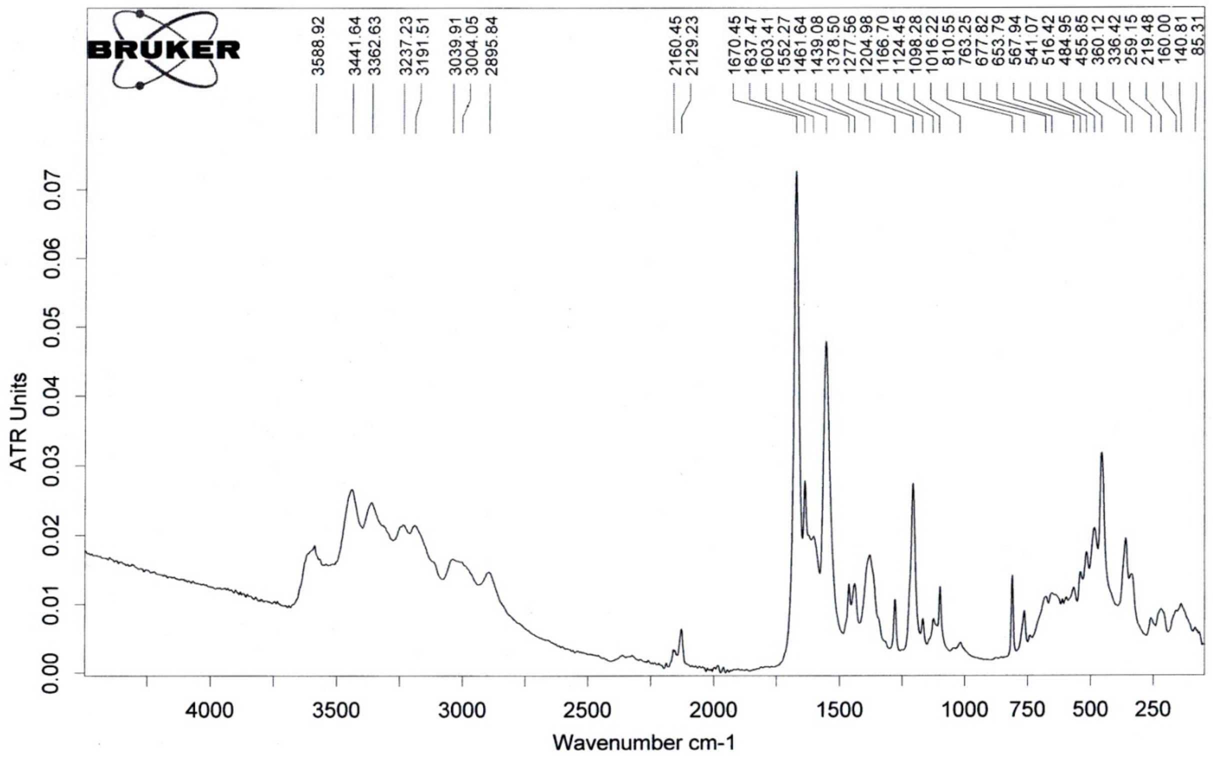

Figure S13. IR spectum of the complex **2.**

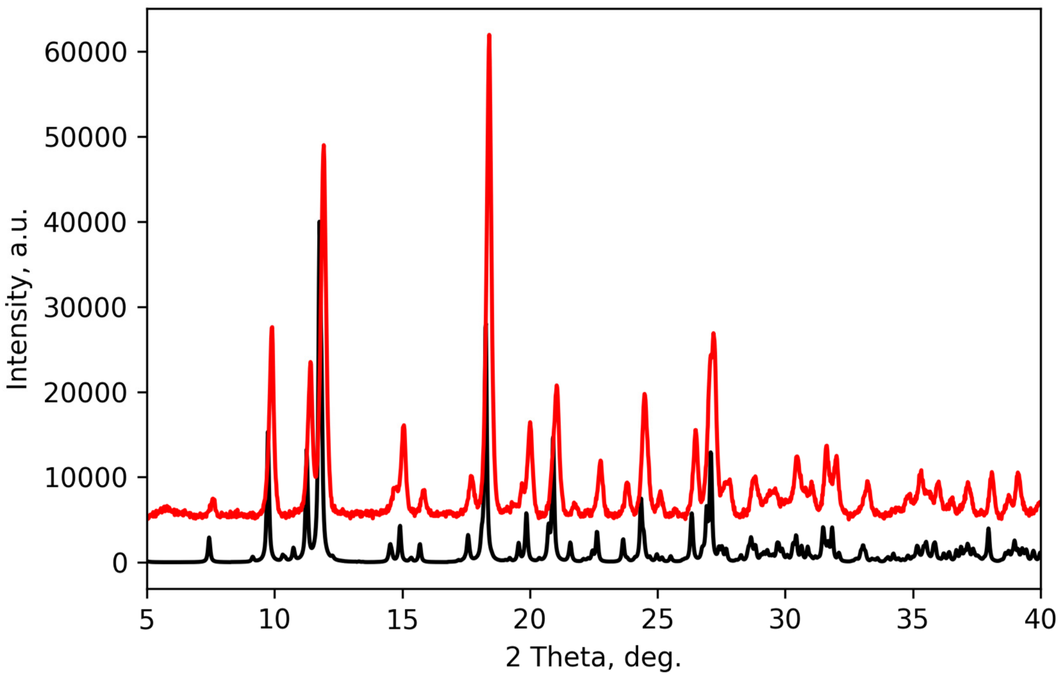

Figure S14. A comparison of the powder diffraction pattern for a dried sample of **2** at 293 K (red) and single crystal simulation of **2** at 120 K (black).

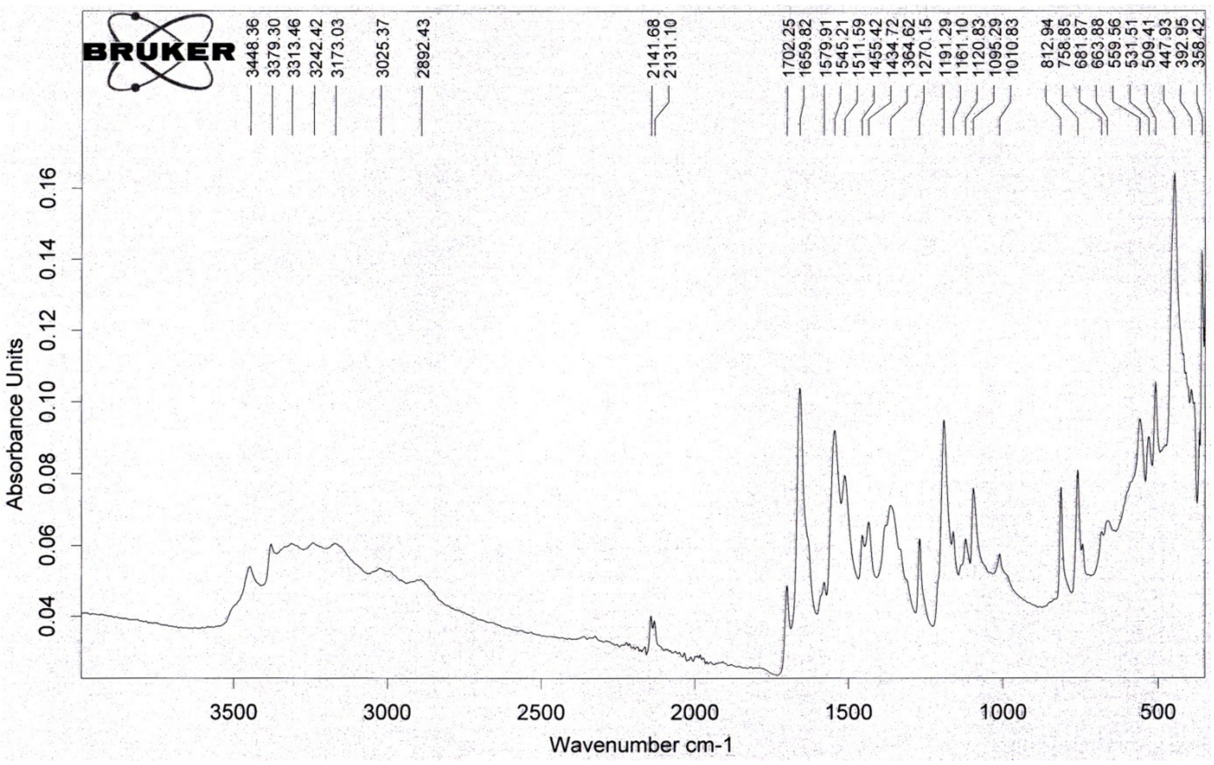

Figure S15. IR spectrum of the complex **3.**

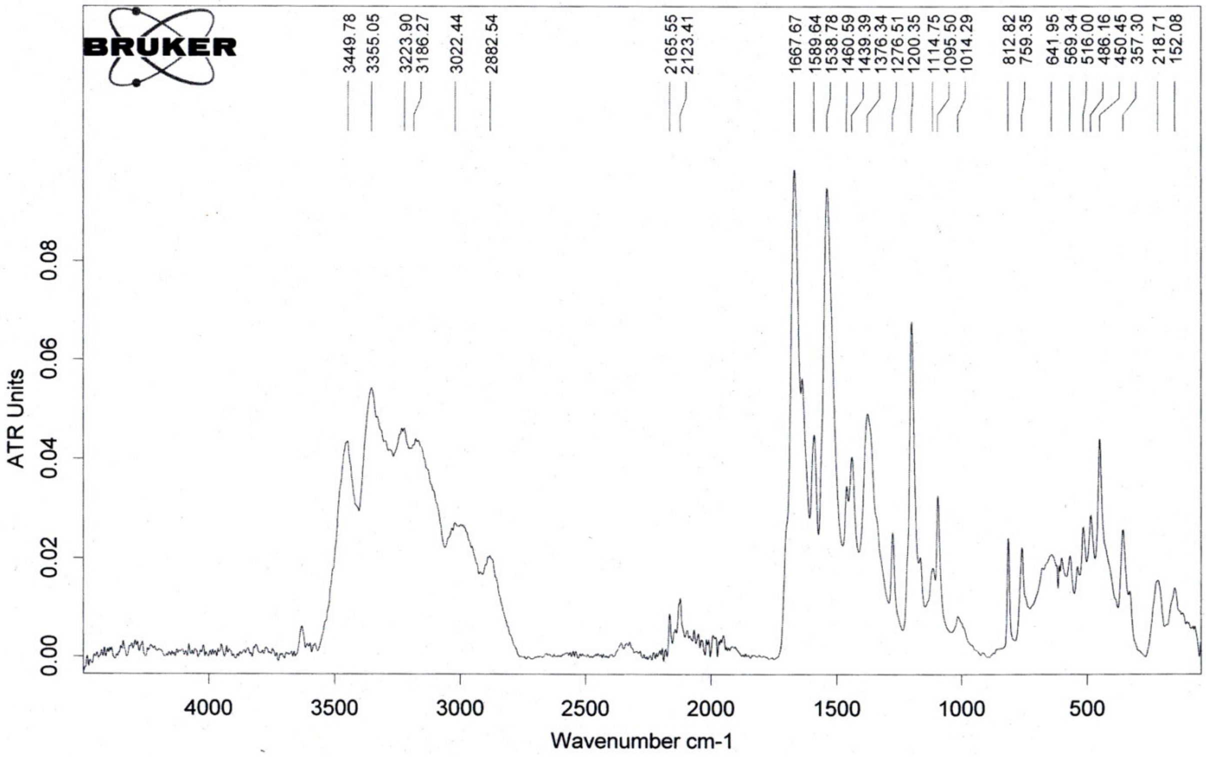

Figure S16. IR spectrum of the complex **4.**
